# Molecular analysis of XPO1 inhibitor and gemcitabine–nab‐paclitaxel combination in KPC pancreatic cancer mouse model

**DOI:** 10.1002/ctm2.1513

**Published:** 2023-12-22

**Authors:** Md. Hafiz Uddin, Mohammad Najeeb Al‐Hallak, Husain Yar Khan, Amro Aboukameel, Yiwei Li, Sahar F. Bannoura, Gregory Dyson, Seongho Kim, Yosef Mzannar, Ibrahim Azar, Tanya Odisho, Amr Mohamed, Yosef Landesman, Steve Kim, Rafic Beydoun, Ramzi M. Mohammad, Philip A. Philip, Anthony F. Shields, Asfar S. Azmi

**Affiliations:** ^1^ Department of Oncology Karmanos Cancer Institute Wayne State University School of Medicine Detroit Michigan USA; ^2^ Detroit Medical Center Detroit Michigan USA; ^3^ UH Seidman Cancer Center University Hospitals, Case Western Reserve University Cleveland Ohio USA; ^4^ Karyopharm Therapeutics Inc. Newton Massachusetts USA; ^5^ Department of Pathology Wayne State University School of Medicine Detroit Michigan USA; ^6^ Henry Ford Cancer Institute Detroit Michigan USA

**Keywords:** digital spatial profiling, gemcitabine, KPC mouse model, nab‐paclitaxel, pancreatic cancer, selinexor, snRNAseq

## Abstract

**Background:**

The majority of pancreatic ductal adenocarcinoma (PDAC) patients experience disease progression while on treatment with gemcitabine and nanoparticle albumin‐bound (nab)‐paclitaxel (GemPac) necessitating the need for a more effective treatment strategy for this refractory disease. Previously, we have demonstrated that nuclear exporter protein exportin 1 (XPO1) is a valid therapeutic target in PDAC, and the selective inhibitor of nuclear export selinexor (Sel) synergistically enhances the efficacy of GemPac in pancreatic cancer cells, spheroids and patient‐derived tumours, and had promising activity in a phase I study.

**Methods:**

Here, we investigated the impact of selinexor–gemcitabine–nab‐paclitaxel (Sel‐GemPac) combination on LSL‐Kras^G12D/+^; LSL‐Trp53^R172H/+^; Pdx1‐Cre (KPC) mouse model utilising digital spatial profiling (DSP) and single nuclear RNA sequencing (snRNAseq).

**Results:**

Sel‐GemPac synergistically inhibited the growth of the KPC tumour‐derived cell line. The Sel‐GemPac combination reduced the 2D colony formation and 3D spheroid formation. In the KPC mouse model, at a sub‐maximum tolerated dose (sub‐MTD) , Sel‐GemPac enhanced the survival of treated mice compared to controls (*p* < .05). Immunohistochemical analysis of residual KPC tumours showed re‐organisation of tumour stromal architecture, suppression of proliferation and nuclear retention of tumour suppressors, such as Forkhead Box O3a (FOXO3a). DSP revealed the downregulation of tumour promoting genes such as chitinase‐like protein 3 (CHIL3/CHI3L3/YM1) and multiple pathways including phosphatidylinositol 3'‐kinase‐Akt (PI3K‐AKT) signalling. The snRNAseq demonstrated a significant loss of cellular clusters in the Sel‐GemPac‐treated mice tumours including the CD44+ stem cell population.

**Conclusion:**

Taken together, these results demonstrate that the Sel‐GemPac treatment caused broad perturbation of PDAC‐supporting signalling networks in the KPC mouse model.

**Highlights:**

- The majority of pancreatic ductal adenocarcinoma (PDAC) patients experience disease progression while on treatment with gemcitabine and nanoparticle albumin‐bound (nab)‐paclitaxel (GemPac).
- Exporter protein exportin 1 (XPO1) inhibitor selinexor (Sel) with GemPac synergistically inhibited the growth of LSL‐KrasG12D/+; LSL‐Trp53R172H/+; Pdx1‐Cre (KPC) mouse derived cell line and enhanced the survival of mice.
- Digital spatial profiling shows that Sel‐GemPac causes broad perturbation of PDAC‐supporting signalling in the KPC model.

## Graphical abstract

The majority of pancreatic ductal adenocarcinoma (PDAC) patients experience disease progression while on treatment with gemcitabine and nab‐paclitaxel (GemPac). Exporter protein exportin 1 (XPO1) inhibitor selinexor (Sel) with GemPac synergistically inhibited the growth of LSL‐KrasG12D/+; LSL‐Trp53R172H/+; Pdx1‐Cre (KPC) mouse derived cell lines and enhanced the survival of mice. Digital spatial profiling shows that Sel‐GemPac causes broad perturbation of PDAC‐supporting signalling in the KPC model.

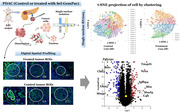

## 1 INTRODUCTION

Pancreatic ductal adenocarcinoma (PDAC) is one of the leading causes of cancer‐associated deaths.^1^ The survival rate of PDAC is dreadfully low.^2^ Due to frequent late diagnosis of the disease, the majority of PDAC patients are not amenable to surgery as a treatment option. For patients with better performance status who are not surgical candidates, FOLFIRINOX is used as first‐line therapy in advanced disease. However, the median overall survival (OS) of metastatic PDAC patients remains dismal, averaging less than a year, even with this highly potent and poorly tolerated chemotherapy combination.^3^ For patients with poor performance status, gemcitabine (Gem) combined with nanoparticle albumin‐bound (nab)‐paclitaxel (GemPac) has been considered as a standard of care first‐line therapy following the MPACT phase III trial published in 2013, where the median OS improved to 8.7 months for GemPac‐treated patients compared to 6.6 months for patients treated with Gem alone (*p* = .001).^4^ Despite a large majority of patients with advanced PDAC receiving GemPac‐based therapy,^5^ most PDAC patients experience disease progression suggesting an urgent need for novel therapeutic strategies.

Exportin 1 or XPO1 is the major nuclear export protein of the cells that is involved in the export of many tumour suppressors from the nucleus to cytoplasm^6^ and is frequently overexpressed in PDAC tumour tissue, indicating its involvement in the progression of PDAC.^7^, ^8^ XPO1 expression has also shown to be associated with tumour size, lymph node invasion, and liver metastasis and can act as a prognostic marker.^9^ The overexpressed XPO1 protein exports many tumour suppressor proteins from the nucleus to the cytoplasm leading to their functional inactivation.^6^ We have demonstrated in our earlier studies that selective inhibitor of nuclear export (SINE) compounds (selinexor—Sel and analogues) synergise with Gem or GemPac and suppress the growth of PDAC cells, spheroids and patient‐derived tumours through global reorientation of proteins in cancer cell nucleus. In a phase I study, durable responses were observed in patients on selinexor–gemcitabine–nab‐paclitaxel (Sel‐GemPac) treatment.^9^, ^10^

Given the multitude of pathways regulated by XPO1, it is critical to take a broader approach for understanding the impact of nuclear export inhibition on PDAC tumour and microenvironment supporting pathways. In the present study, we have tested the Sel‐GemPac combination in the KPC pancreatic cancer mouse model and assessed the broader tumour microenvironment utilising digital spatial profiling (DSP) and single nuclear RNA sequencing (snRNAseq) approaches.

## 2 MATERIALS AND METHODS

### 2.1 Cell lines and reagents

LSL‐KrasG12D/+; LSL‐Trp53R172H/+; Pdx1‐Cre (KPC)‐derived tumour cell line KCI‐313 was established in the laboratory following standard procedure and maintained in Dulbecco's modified Eagle medium.^11^ The medium was supplemented with foetal bovine serum (10%) and penicillin and streptomycin (1%), and the cells were kept at 37°C in a humidified condition with 5% CO_2_ (Stericycle CO_2_ incubator, Thermo Scientific). The cell line was authenticated (STR profiling) and ‐ by polymerase chain reaction (PCR) and tested negative for *Mycoplasma* spp. Ki‐67 (catalogue no. M7240) and CD4 (catalogue no. M731029‐2) antibodies were purchased from Dako. Forkhead Box O3a (FOXO3a) (catalogue no. 12829) antibody was purchased from Cell Signalling Technology. CHIL3 (catalogue no. PA5‐81356) and F4/80 (catalogue no. MA1‐91124) were purchased from ThermoFisher Scientific. Horseradish peroxidase (HRP)‐conjugated secondary antibody Pollink‐2 Plus HRP Broad for DAB Kit (catalogue nos. D41‐110 and D35‐6) was used (Golden Bridge International [GBI] Labs).

### 2.2 KPC mouse model

The KPC mouse model of spontaneous PDAC was bred as described previously.^12^ The animal protocol used in the study was approved by Wayne State University Institutional Animal Care and Use Committee (IACUC# 20‐07‐2492). These mice express activated Kras and mutated Trp53 in the developing pancreas conditionally and spontaneously develop sequential ductal lesions from pancreatic intraepithelial neoplasias (PanINs) to metastatic PDAC. This model almost accurately mimics the human disease from initiation to progression. The mice were crossed and bred in the animal facility of Wayne State University. Mouse DNA was isolated from the ear using the Wizard Genomic DNA Purification Kit (Promega), as per the manufacturer's protocol. The ear tissue was digested overnight with continuous agitation and genomic DNA was extracted. To confirm the pdx1‐Cre introduction and mutations of KRAS and p53, conventional PCR was performed. Four to 5‐week‐old genotyped and confirmed KPC mice were enrolled in two groups on a rolling basis. Mice were randomly allotted into two groups: control (*n* = 13) and treatment (*n* = 14). The control group received the vehicle and the treated group received the Sel‐GemPac combination, where Sel was given orally at 15 mg/kg twice a week for 4 weeks and Gem and nab‐Pac were given intravenously (i.v.) at 30 mg/kg twice a week for 2 weeks. During treatments, mice were followed daily for any signs of distress from either treatment or illness due to tumour growth; however, mice were not examined for the presence of tumour. After euthanasia, a gross pathological examination was performed. The survival curve was generated using GraphPad Prism (version 5.0). A few mice were examined for the development of tumours in pancreas using high frequency ultrasonography. Prior to imaging, a small area (1–2 cm in diameter) on the left abdominal flank was shaved. Mice were anaesthetised using isoflurane. Once adequate anesthesia was confirmed with no reflex noted, a small amount of warm Aquasonic ultrasound gel (warmed using Thermosonic Gel Warmer) was placed on the shaved site. Using a single element probe (PB506e; 30−60 MHz) directly on the gel, organ/tumour images were acquired (Scintica).

### 2.3 Single nuclear RNA sequencing

Randomly selected tumours from KPC mice with or without treatment (*n* = 2 each group) were harvested and snRNAseq was performed in a blinded fashion (Singulomics Corporation; http://singulomics.com/). Briefly, tumour cell nuclei were isolated from frozen mouse pancreatic cancer tissue samples. The tissue was subsequently subjected to homogenisation and lysis using Triton X‐100 in RNase‐free water to facilitate the isolation of nuclei. The purified nuclei were further processed, undergoing centrifugation, resuspension in phosphate‐buffered saline (PBS) containing RNase inhibitor and then diluted to a concentration of 700 nuclei/μL to enable a standardised 10× Genomics capture. For the two control samples, 6565 and 3093 cell nuclei were filtered and for two treated samples, 6800 and 3366 cell nuclei were filtered out, where at least 50 genes were detected. The 3′ single cell gene expression libraries (Next GEM v3.1) were constructed using the 10× Genomics Chromium system. The libraries were sequenced with ∼400 million PE150 reads per sample on Illumina NovaSeq. After sequencing, clean reads were analysed with mouse reference genome mm10 using Cell Ranger (version 6.0.1). Introns were included in the analysis and aggregation of the samples was also performed. Principal component analysis was performed to reduce the number of feature dimensions. The t‐distributed stochastic neighbour embedding (t‐SNE) analysis was performed to visualise cells in a 2D space and clustering of cells that have similar expression profiles.

### 2.4 Growth inhibition assay

The growth inhibition was determined by 3‐(4,5‐dimethylthiazol‐2‐yl)−2,5‐diphenyltetrazolium bromide (MTT) assay. In brief, KCI‐313 cells were plated onto 96‐well plates at a density of 4000 cells/well. The cells were maintained for 72 h in the presence of drugs. After treatment cells were treated with MTT for 2 h and formazan crystals were dissolved in dimethyl sulphoxide, the optical density was measured at a wavelength of 570 nm using a spectrophotometer. IC_50_ values were determined from GraphPad Prism 5 software and the closest round number of IC_50_ doses was used.

### 2.5 Immunohistochemical analysis

Immunohistochemical (IHC) analysis of KPC residual tumour tissues for Ki‐67, FOXO3a, CHIL3 and CD4 was performed in the histopathology core facility of Karmanos Cancer Institute, Wayne State University, in a double‐blinded fashion. For IHC analysis, all the tumour tissues from KPC mice were stored in 10% neutral‐buffered formalin (v/v). Standard procedures were followed for IHC. Briefly, paraffin sections were de‐waxed and rehydrated in a xylene–ethanol series. Endogenous peroxides were removed by a methanol/1.2% hydrogen peroxide solution and incubation at room temperature for 25 min. Heat‐induced epitope retrieval was performed with a pH 6.0 citrate buffer (Ki‐67) and pH 9.0 ethylenediaminetetraacetic acid (EDTA) buffer (CD4 and FOXO3a) using the BIOCARE Decloaking Chamber. Before adding the primary antibody, a 40‐min blocking step was conducted using Super‐Block Blocking buffer (Thermo Scientific). The primary antibody dilutions were 1:50, 1:100, 1:200 and 1:300 for CD4, Ki‐67, FOXO3a and CHIL3, respectively. IHC was performed using GBI Labs DAB chromogen kit (DAB Chromogen Kit). Subsequently, the sections underwent a dehydration process through a series of ethanol to xylene washes before being cover‐slipped. Routine haematoxylin and eosin (H&E) staining was also performed to observe tissue histology. For Picrosirius staining, Picro Sirius Red stain kit (catalogue no. ab150681) from Abcam was used according to manufacturer instructions to visualise collagen distribution. Images were captured at 20× or 40× magnifications. For scoring, the images were scanned using the Aperio CS2 Scanner. Quantification was done with Indica Lab Halo software. The selected algorithm settings used were CytoNuclear v2.0.9 and Membrane v1.7.

### 2.6 Colony formation assay

KPC‐derived tumour cells (100 cells/well) were seeded in 24‐well plates and treated the next day with indicated doses of Gem, nab‐Pac, Sel or their combinations. After 72 h, treatment was withdrawn. Ten days later, colonies were rinsed with PBS and then immersed in 100% methanol for fixation. Afterwards, the colonies were subjected to staining with .5% crystal violet for 20 min and cautiously washed using tap water. Colonies were dried before scanning.

### 2.7 Spheroid formation assay

KPC‐derived tumour cells (100 cells/well) were seeded in triplicate in ultralow attachment 96‐well plates and treated the next day with Gem, nab‐Pac and Sel at 5, 1.8 and 100 nM concentrations, respectively. Three‐dimensional spheroid medium was used with supplements to facilitate the spheroid formation (catalogue no. C‐28077; PromoCell GmbH). Seven days later, spheroids were imaged using a phase contrast microscope fitted with the camera (Olympus). NIH ImageJ software was used to measure the area of the spheroids.

### 2.8 DSP of KPC mice‐derived PDAC at a transcripto‐proteomic scale

DSP was performed on formalin fixed paraffin embedded (FFPE) samples according to the manufacturer's instructions (NanoString Technologies). In brief, the 5 μM tissue sections from two control and two treated mouse tumours were affixed in the middle of a charged slide. The slide was baked at 60°C for 30 min and subjected to deparaffinisation, rehydration and antigen retrieval. To perform RNA profiling, a set of probes designed for mRNAs (Mouse Whole Transcriptome Atlas RNA v1.0) was applied to the sample and allowed to hybridise at 37°C overnight inside a hybridisation oven. In preparation for protein profiling, subsequently, a tricolour fluorescence morphology marker panel was applied to the slides, targeting pan‐cytokeratin (Pan‐CK; 2 μg/mL; Novus Biologicals; catalogue no. NBP2‐33200) for epithelial and tumoural regions, CD45 (1:40 dilution; Novus Biologicals; catalogue no. NBP1‐44763AF594) for immune cells and SYTO83 (.2 μM; ThermoFisher Scientific; catalogue no. S11364) for the nucleus. Specific oligo‐conjugated primary antibodies cocktails, known as the Pan‐Tumour panel, were incubated with the slides. Every target probe is equipped with an exclusive photocleavable oligonucleotide barcode, which is amenable to quantification through next‐generation sequencing. For both RNA and protein profiling, the slide is loaded into the profiler covered with an acquisition buffer. The utilisation of multicoloured morphology markers enables the visualisation of different compartments. This visualisation aids in guiding the selection of regions of interest (ROIs). After the one‐step overnight incubation, the samples underwent 20× high‐precision scanning using a GeoMx DSP system, followed by the selection of ROIs. The DNA oligonucleotides, which were attached to the profiling reagents, are released sequentially using ultraviolet illumination and gathered into individual wells on a 96‐well plate. Subsequently, the collected DNA is subjected to Illumina library preparation. The expression levels are measured using an Illumina Sequencer and then analysed using the DSP interactive software (GeomxTools, version 1.99.4; Advanced Genomics Core). In RNA profiling, a total of 20,172 probes passed all thresholds. The raw read counts per gene, which were imputed, underwent normalisation using the third quartile (Q3) method. Q3 normalisation was applied on the filtered dataset of 11,666 target genes.

### 2.9 Analysis with iPathwayGuide

We have utilised iPathwayGuide (version 2201) to perform pathway analyses (Advaita Bioinformatics; http://advaitabio.com/ipathwayguide).^13^ The downstream analysis utilised the normalised fold‐change (FC) and *p*‐values for all expressed genes as its input. Pathways were ranked using the impact analysis method.^14^, ^15^ Enrichment analysis of gene function and cellular pathways was performed for differentially expressed genes (DEGs) using the same software with the default *Mus musculus* data as background. We performed pathway enrichment analysis on the set of genes that were upregulated by Sel‐GemPac in cancer or stromal cells. For Gene Ontology (GO) analysis, an over‐representation test, based on a hypergeometric distribution, was used to compute the statistical significance of observing more than the expected number of DEG. Pathway over‐representation analysis was performed by comparing the number of affected genes associated with a pathway between groups.

### 2.10 Real‐time reverse transcriptase quantitative PCR

Real‐time reverse transcriptase quantitative PCR (RT‐qPCR) was performed as described earlier.^16^ The primers utilised in this work are presented in Table S1. Primer sets for XPO1 and GAPDH were designed using a primer designing tool from NCBI (https://www.ncbi.nlm.nih.gov/tools/primer‐blast/). Primer sets for COL4A2 and C4B were selected from Primerbank (https://pga.mgh.harvard.edu/cgi‐bin/primerbank/).

### 2.11 Immunoblot analysis

Whole‐cell lysate preparation and immunoblot were performed using RIPA lysis buffer system (Santa Cruz Biotechnology; catalogue no. sc‐24948) and standard western blot technique. The protein concentration was measured using bicinchoninic acid (BCA) protein assay (ThermoFisher Scientific). A total of 40 μg protein lysate was loaded and separated using 10% sodium dodecyl sulfate‐polyacrylamide gel electrophoresis (SDS‐PAGE). The proteins were transferred to nitrocellulose membrane (.45 μM) and blocked with 3% bovine serum albumin in PBS with .1% Tween‐20. The primary antibody dilution was 1:1000. LI‐COR goat anti‐mouse (IRDye 800CW; catalogue no. 827‐08364) and goat anti‐rabbit (IRDye 680RD; catalogue no. 926‐68171) secondary antibodies were used for the detection. Signals were detected and images were visualised using Odyssey infrared imaging system (Odyssey DLX, LI‐COR Biosciences).

### 2.12 Statistical analysis

The in vitro experiments were done at least in triplicate. Data are represented as mean ± standard deviation or standard error of mean. The data were compared using Student's *t*‐test. Survival between control and treated mice was compared using the log‐rank (Mantel–Cox) test. For in vivo experiments, duplicate samples were subjected to snRNAseq or DSP analysis. The *p*‐value of <.05 was considered as statistically significant. To identify differentially expressed proteins in volcano plots, FC of 1.5 and unadjusted *p*‐value of .05 were used. A GO term or pathway was considered statistically significant at false discovery rate (FDR) corrected at *p* = .05 (^*^ *p* < .05, ^**^ *p* < .01 and ^***^ *p* < .001).

## 3 RESULTS

### 3.1 Synergistic effect of Sel‐GemPac combination in KPC tumour‐derived KCI‐313 cell line

**FIGURE 1:**
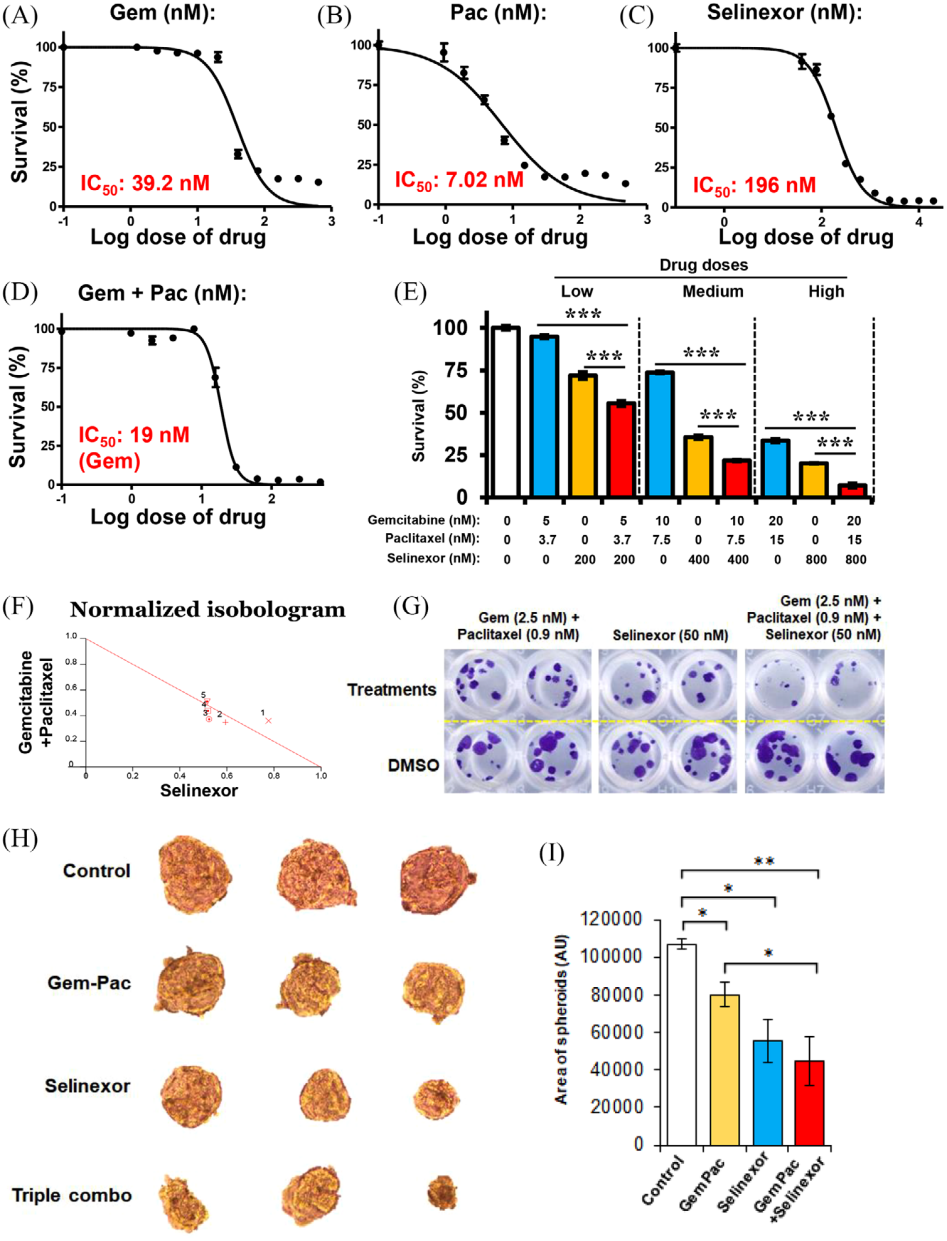
Effect of selinexor–gemcitabine–nab‐paclitaxel (Sel‐GemPac) combination in LSL‐Kras^G12D/+^; LSL‐Trp53^R172H/+^; Pdx1‐Cre (KPC) tumour‐derived KCI‐313 cell line. (A–E) Growth inhibition of KCI‐313 cells after gemcitabine (Gem), paclitaxel (Pac), selinexor (Sel) and combination treatments determined by 3‐(4,5‐dimethylthiazol‐2‐yl)−2,5‐diphenyltetrazolium bromide (MTT) assay. Dimethyl sulphoxide (DMSO) or drug combination treatments were done for 72 h. (A–C) The log_10_ dose–response curves with determined IC_50_ values for Gem, Pac and Sel. (D) The log_10_ dose–response curves with determined IC_50_ values for Gem when treated with Pac. (E) The combination of gemcitabine–nab‐paclitaxel (GemPac) and Sel with three different doses. All the doses are shown in nM concentrations. Combination treatments were compared to GemPac or Sel only using Student's *t*‐test. (F) Normalised isobologram for Gem, Pac and Sel combination treatments were generated using Calcusyn 2.0 software. (G) Colonies from KCI‐313 cells treated with indicated doses of Gem, Pac, Sel and their combinations. After 72 h cells were maintained in drug free media for ten additional days. Colonies were fixed and stained with .5% crystal violet solution containing methanol. (H) Three‐dimensional spheroid formation assay. KCI‐313 cells were seeded in an ultra‐low attachment plate with serum reduced media supplemented with growth factors. After a week of treatment, spheroids were imaged and analysed for their area using NIH ImageJ software (I). ^*^ *p* < .05; ^**^ *p* < .01; ^***^ *p* < .001.

The characterisation of the KCI‐313 cell line is detailed in Figure S1. We tested each drug alone and in combination in the KPC‐derived cell line and observed a dose‐dependent effect on growth inhibition (Figure 1A–E). The analysis of data with Calcusyn 2.0 revealed several synergistic combinations among Sel, Gem and Pac, which is evident from normalised isobologram (Figure 1F). To validate the effect of combination, we further tested it on the colony forming ability of the KCI‐313 cells. Surprisingly, a very low concentration of these drugs (Sel 50 nM, Gem 2.5 nM and Pac .9 nM) was able to suppress the colony forming ability of KCI‐313 cells (Figure 1G). The Sel‐GemPac triple combination not only reduced the colony number but also reduced the size of the colonies. These 2D results were corroborated in the 3D spheroid model system (Figure 1H,I). The area of the spheroids was significantly reduced in the triple combination compared to control and GemPac alone. Additionally, a trend towards smaller spheroids was observed in the triple combination‐treated colonies when compared to the Sel only group.

### 3.2 Sel‐GemPac treatment enhanced the survival of KPC mouse model

**FIGURE 2:**
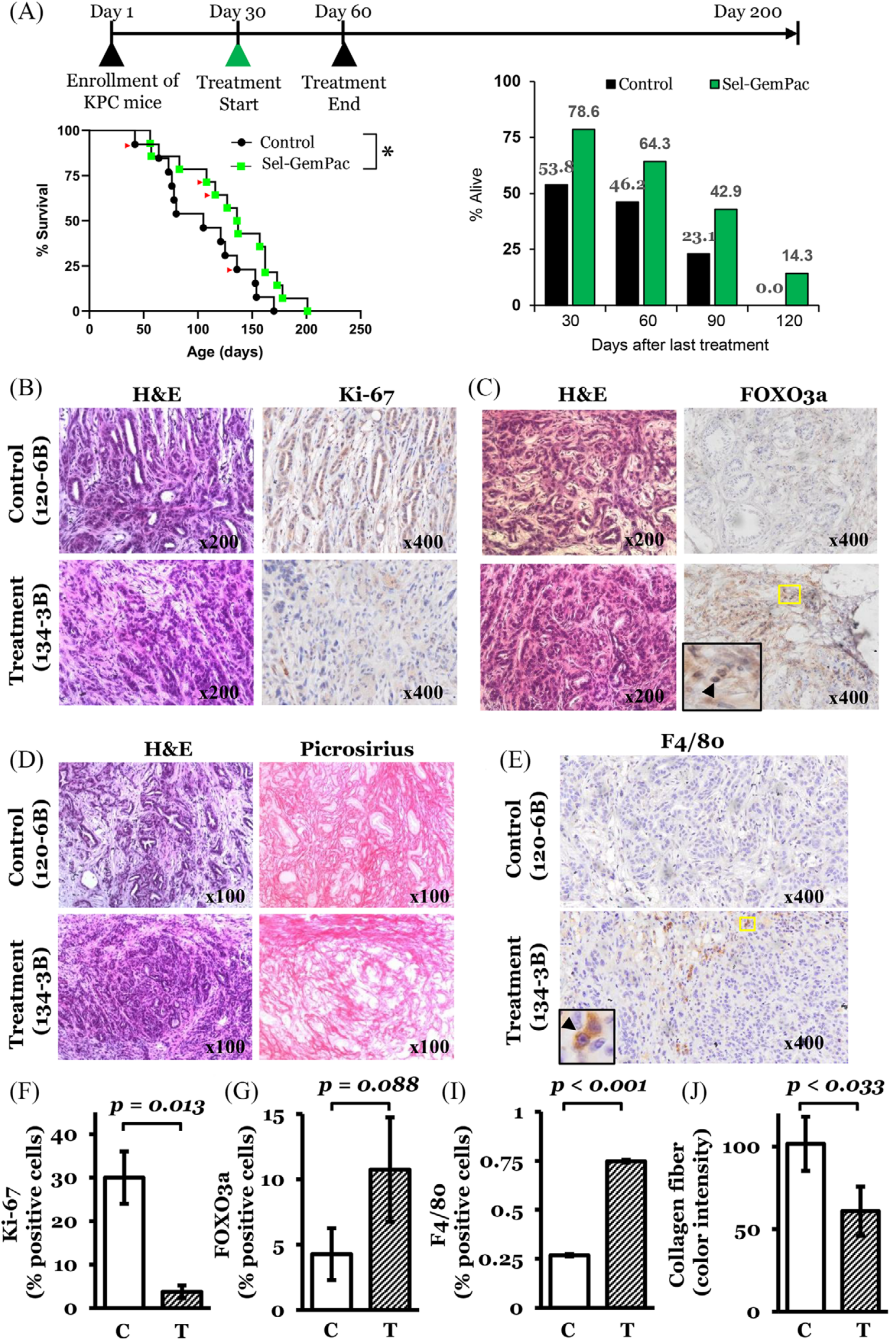
Survival and immunohistochemical analysis of selinexor–gemcitabine–nab‐paclitaxel (Sel‐GemPac)‐treated LSL‐Kras^G12D/+^; LSL‐Trp53^R172H/+^; Pdx1‐Cre (KPC) mice tumours. (A) Top: Treatment plan. Left: Kaplan–Meier survival plot for control (*n* = 13) and Sel‐GemPac (*n* = 14)‐treated KPC mice. Selinexor (Sel) was given at a dose of 15 mg/kg orally (2×/week ×4). Gemcitabine (Gem) and paclitaxel (Pac) were given at a dose of 30 mg/kg i.v. twice a week for 2 weeks. Red arrow heads indicate the mice used for further analysis. Right: Percentage of survived mice at different time points after last treatment in control and Sel‐GemPac groups. (B and C) Immunohistochemical staining of Ki‐67 and Forkhead Box O3a (FOXO3a) in representative control (120‐6B) and treated (134‐3B) tumour tissues, respectively. Nuclear accumulation of FOXO3a is enlarged and marked with arrowhead (lower panel). (D) Picrosirius staining performed on representative control (120‐6B) and treated (134‐3B) tumour tissues. Corresponding haematoxylin and eosin (H&E) staining is shown in the left panel. Original magnifications are shown on each histopathological image. (E) F4/80 immunohistochemical staining. (F–I) Percentage of Ki‐67, FOXO3a and F4/80‐positive cells in the tissue samples. (J) Abundance of collagen fibre measured as colour intensity by NIH ImageJ software. C, control; T, Sel‐GemPac treated. ^*^ *p* < .05.

Next, we tested the efficacy of Sel‐GemPac in the KPC mouse model. All KPC mice were genetically confirmed to carry Kirsten rat sarcoma virus (*KRAS*) and tumour protein P53 (*p53*) mutations by PCR (Figure S2A,B) and randomly assigned to control and treated groups. An ultrasonographic examination was performed to visualise suspected tumour in the pancreas (Figure S2C). Histopathological examination has confirmed the tumours as PDAC (Figure S2D). Poorly differentiated adenocarcinoma is evident in the histopathological section. A total of 13 KPC mice in the control group and 14 KPC mice in the treatment group were used for the survival analysis. An increase in median survival by 1 month was observed in the Sel‐GemPac‐treated mice (median survival 105 and 136.5 days for control and Sel‐GemPac‐treated groups, respectively). Approximately, 78.6% of the mice survived in the Sel‐GemPac treatment group just after 30 days of last treatment, compared to only 53.8% in the control group (Figure 2A). After 120 days of drug treatment, survival rates were 0% and 14.3% in the control and treated groups, respectively. The difference in the OS between control and Sel‐GemPac‐treated group was statistically significant (*p* = .047, log‐rank [Mantel–Cox] test). The findings indicate that the Sel‐GemPac therapy could prolong the survival of KPC mice.

### 3.3 IHC and histochemical analyses of Sel‐GemPac‐treated KPC tumour tissues confirms the anti‐proliferative role of this therapy

IHC analysis revealed lower expression of the proliferation marker Ki‐67 in the Sel‐GemPac‐treated KPC mice tumour tissues (Figure 2B,F). The reduced Ki‐67 expression indicates a growth suppressive role of this combination in the KPC mouse model. FOXO3a, a recognised nuclear export cargo of exportin 1 (XPO1), is a transcription factor that has been shown to play a significant role in the progression of various cancers, including PDAC. Its expression is often downregulated and associated with poor prognosis in PDAC.^17^ Our results showed both an increase in the expression and nuclear accumulation of FOXO3a tumour suppressor in drug‐treated KPC tissues (Figure 2C). The score shows an increasing trend even though not to the point of significance (Figure 2G). This finding provides further support that Sel‐GemPac activity in PDAC growth inhibition is partly due to the nuclear retention of FOXO3a.

Collagen plays a significant role in the progression of PDAC and metastasis. PDAC is characterised by a dense desmoplastic stroma and consists of abundant collagen fibres. The collagen‐abundant stroma offers a supportive microenvironment for cancer cells to grow and invade.^18^ We performed picrosirius staining to demonstrate the level and architecture of collagen in the Sel‐GemPac‐treated KPC mice tumours compared to control tumours. Our results show a lower level of collagen fibres in the treated mice tumour tissue along with evidence of collagen degradation (Figure 2D). The abundance of collagen fibre was measured as colour intensity and found to be significantly low in Sel‐GemPac‐treated tumour tissue (*p* < .05) (Figure 2J). F4/80 staining was significantly higher (*p* < .001) in the treated sample suggesting a robust immune response in the tissue (Figure 2E,I). Collectively, these findings point to the role of Sel‐GemPac in PDAC microenvironmental modulation.

### 3.4 DSP of transcriptome identified DEGs and pathways in Sel‐GemPac‐treated KPC mice tumour tissues

**FIGURE 3:**
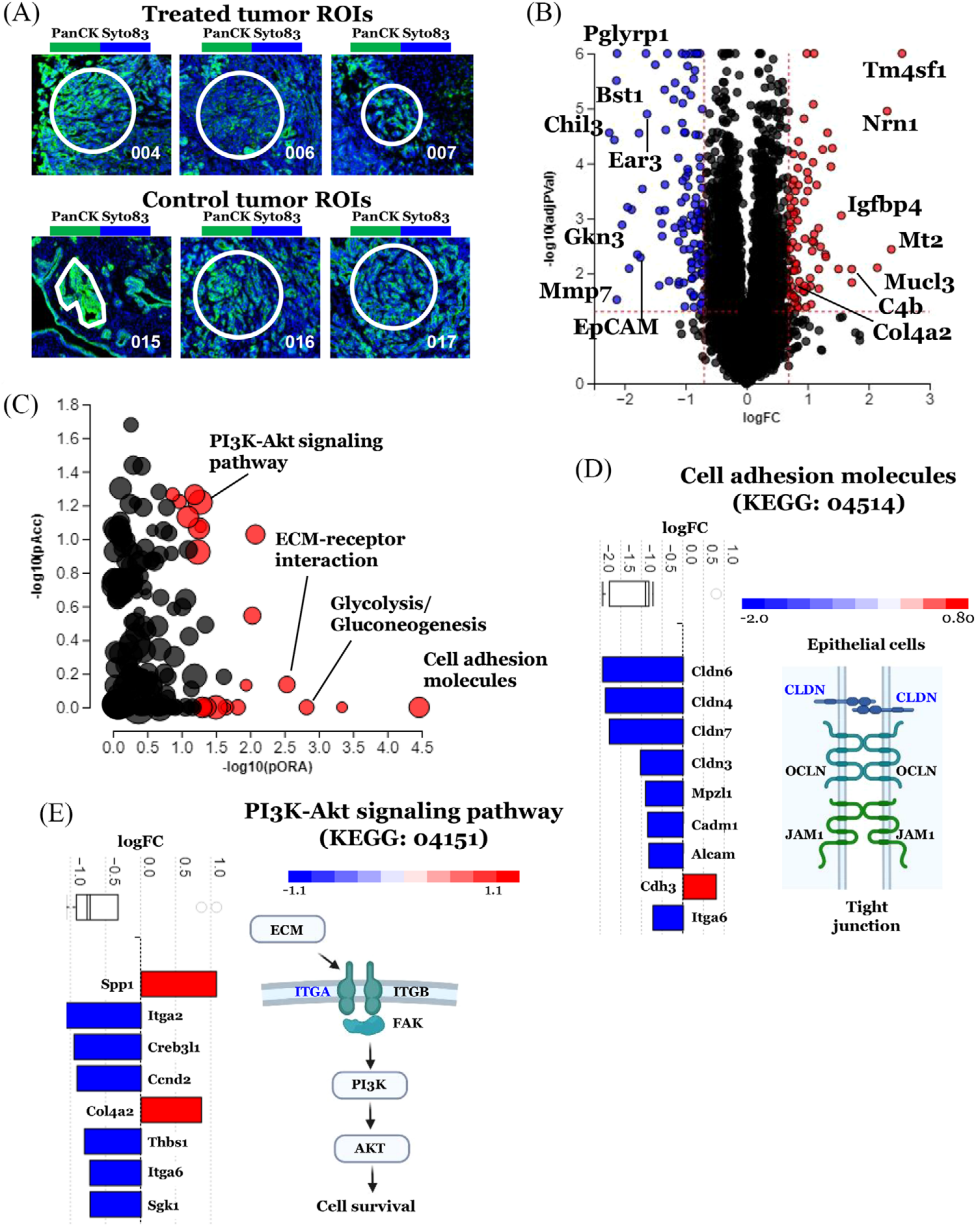
Digital spatial transcriptomics (DSP) analysis of control and treated LSL‐Kras^G12D/+^; LSL‐Trp53^R172H/+^; Pdx1‐Cre (KPC) mice tumours. (A) Selected three region of interests (ROIs) from treated and control KPC mice tumours containing more than 50 cells. For the selection of specific cell types, circle, rectangle or irregular shapes were drawn around the regions. Morphology markers mouse pan‐cytokeratin (panCK, green) and CD45 (yellow) were used to distinguish cancer cells and immune cells . DNA was stained with SYTO83 dye (blue). ROIs were selected in triplicates based on haematoxylin and eosin (H&E) staining and morphology markers. (B) Differentially expressed genes (DEGs) between treated and untreated KPC mice tumour sections. The dotted lines are used to select the DEGs. Significantly (*p* < .05) overexpressed genes are shown in red and under‐expressed genes are shown in blue. (C) Top impacted pathways in the treated tumours compared to control tumours. The *x*‐axis indicated over‐representation (pORA) and the *y*‐axis indicated total pathway accumulation (pAcc). Each dot represents a pathway and the dot size is proportional to the represented pathway. Significant and non‐significant pathways are shown in red and grey, respectively. (D and E) Impacted genes and pathways associated with cell adhesion molecules and phosphatidylinositol 3'‐kinase‐Akt (PI3K‐AKT signalling pathway). The genes that show differential expression are arranged according to their log fold‐change (FC). Genes that have been upregulated are visualised in red, while genes that have been downregulated are represented in blue. On the top, the box and whisker plot provide a summary of the distribution of all genes. The box in the plot depicts the first quartile, median and third quartile of the distribution, whereas any outliers in the data are depicted as circles. Each gene's computed perturbation is overlaid on the pathway diagram (text colour). The perturbation considered both the measured FC of each gene and the accumulated perturbation propagated from any upstream genes, accounting for the cumulative effect on downstream genes. The highest negative and positive perturbations are shown in dark blue and dark red, respectively.

Next, we performed DSP of KPC mice tumour tissue sections utilising GeoMx nanostring technology (NanoString Technologies). The cancer cells in the treated and control tumour sections were precisely marked as ROIs based on H&E staining and mouse Pan‐CK morphology marker (Figure 3A). The application of IHC staining for mouse immune marker CD45, facilitated the selection of cancer cells in both groups via exclusion of immune cells. The use of DNA staining enabled identifying an adequate quantity of cells for examination. Identification of approximately 12,000 transcripts from each selected ROI was made possible by using photocleavable complementary oligonucleotides. Subsequent analysis has determined DEGs between cancer cell ROIs of treated and control KPC tissue samples. In this experiment, 197 DEGs were identified out of a total of 11,650 genes with measurable expression. Among them, 82 genes were upregulated, and 115 genes were downregulated (cutoff for FDR = .05). Volcano plot showed significantly upregulated transcripts of transmembrane 4 L6 family member 1 (*TM4SF1*), neuritin 1 (*NRN1*), *MT2*, mucin‐like protein 3 (*MUCL3*), complement component 4B (*C4B*) and insulin‐like growth factor‐binding protein 4 (*IGFBP4*) genes in treated KPC mice cancer cells. On the other hand, transcripts of *CHIL3*, bone marrow stromal cell antigen 1 (*BST1*), peptidoglycan recognition protein 1 (*PGLYRP1*), gastrokine 3 (*GKN3*) and matrix metalloproteinase‐7 (*MMP‐7*) genes were significantly downregulated in treated KPC mice cancer cells (Figure 3B and Table S2 for all DEGs). A similar comparison was performed between stromal cell ROIs of treated and control KPC mice tumour tissue samples (Figure S3). A total of 42 DEGs were identified out of 11,650 genes with measured expression (Table S3). Among the 42 genes captured, 14 genes were upregulated, and 28 genes were downregulated (cutoff for FDR = .05). The collagen type IV alpha 2 chain (*COL4A2*) gene simultaneously overexpressed in the cancer and stromal compartment of treated KPC mice tumour tissue. Conversely, multiple genes such as *CHIL3*, epithelial cellular adhesion molecule (*EpCAM*), apolipoprotein E (*APO‐E*), COUP transcription factor 1 (*COUP‐TF1* or *EAR3*), midline‐1 (*MID1*), extracellular matrix protein 1 (*ECM1*) and *GKN3* were found to be under‐expressed simultaneously in the cancer cells and stroma of treated KPC mice tissue. These findings imply Sel‐GemPac could influence pathways both in stroma and cancer cells in KPC mouse tumours.

Using iPathwayGuide, we have scored the impacted pathways, which uses impact analysis method to score pathways via the over‐representation of DEGs and the perturbation computed by propagating the measured expression changes across the pathway topology.^13^ Among the 16 pathways that were significantly impacted, we identified cell adhesion molecules (CAM), ECM‐receptor interaction, glycolysis/gluconeogenesis and PI3K‐AKT signalling as the most affected pathways in the treated tumours when compared to the control tumours (Figure 3C). CAM are expressed on the surface of cells and are critical in a diverse range of biological processes, including inflammation.^19^ Out of 96 CAM genes, nine genes including claudin (*CLDN‐3*, *‐4*, *‐6*, *‐7*), myelin protein zero like 1 (*MPZL1*), cell adhesion molecule 1 (*CADM1*), cadherin 3 (*CDH3*), activated leukocyte cell adhesion molecule (*ALCAM*) and integrin alpha‐6 (*ITGA6*) were significantly modulated in the cancer cells of treated tumours. All the genes except CDH3 are downregulated suggesting a negative regulation of this pathway. The negative regulation could affect the tight and other junctions of epithelial cells through downregulated *CLDN* genes (Figure 3D, right panel). There were eight DEGs in PI3K‐AKT signalling pathway. Among these DEGs, six were downregulated, which included ITGA6. The ITGA6 may negatively regulate ECM‐induced PI3K‐AKT signalling (Figure 3E) thereby could suppress the survival of cancer cells. Collectively, our results indicate that Sel‐GemPac treatment could improve the survival of KPC mice through broad modulation of tumour and stroma‐associated pathways.

### 3.5 GO analysis from DSP transcriptomics data identified key processes and functions in Sel‐GemPac‐treated KPC mice tumour tissues

**FIGURE 4:**
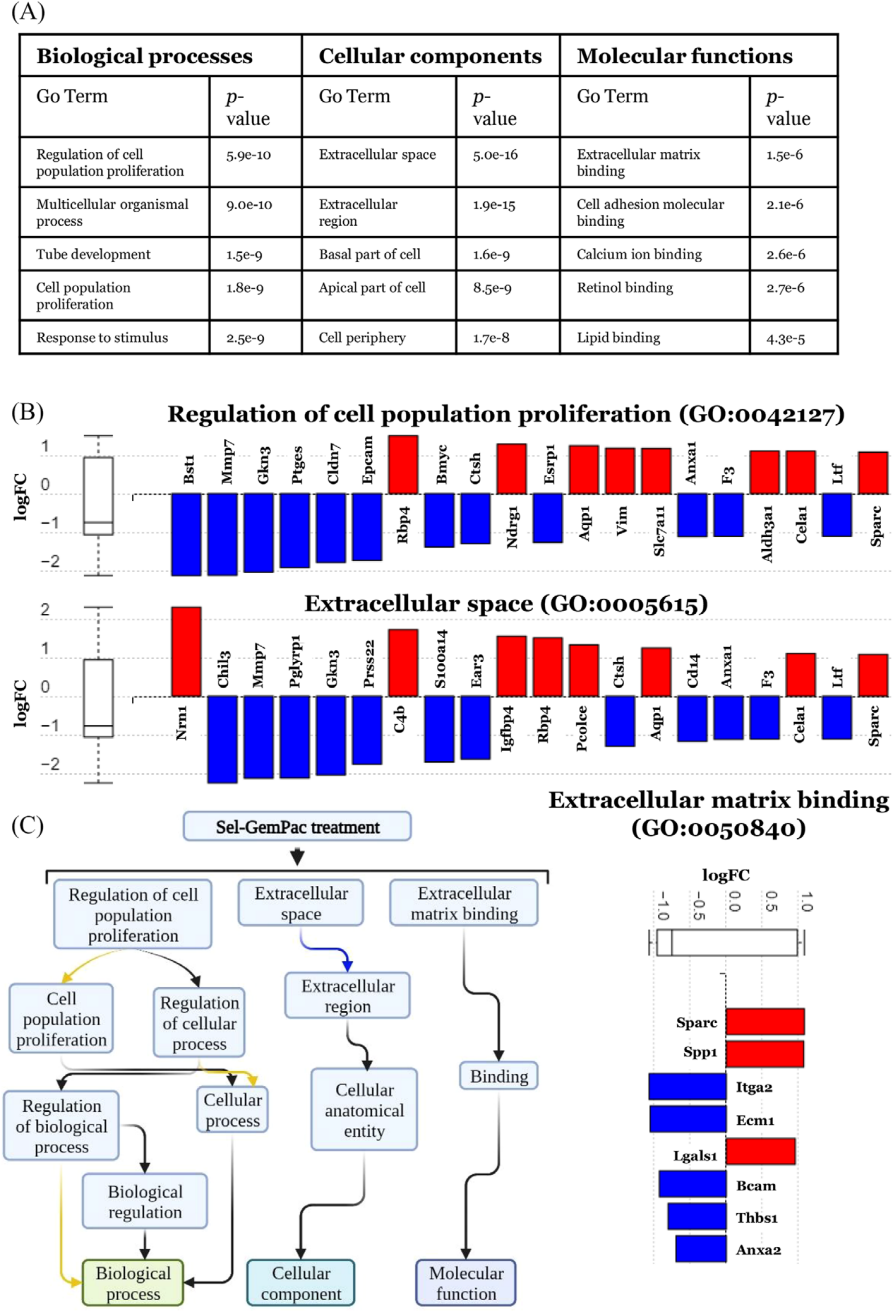
Gene Ontology (GO) analysis of control and treated LSL‐Kras^G12D/+^; LSL‐Trp53^R172H/+^; Pdx1‐Cre (KPC) mice tumours. (A) The top identified biological processes, cellular processes and molecular functions. The number of genes that are differentially expressed and associated with a particular GO term is compared to the expected number of differentially expressed genes (DEGs) for that term just by chance. An over‐representation approach (pORA) is used by iPathwayGuide to compute the statistical significance of observing at least the given number of DEGs. The hypergeometric distribution is utilised to calculate the p‐value, which is then adjusted for multiple comparisons using false discovery rate (FDR). (B) Gene measured expression bar plots. Gene expression analysis of cancer tissues performed between selinexor–gemcitabine–nab‐paclitaxel (Sel‐GemPac)‐treated KPC mice compared to untreated KPC mice. All the DEGs involved in regulation of cell population proliferation, extracellular space and extracellular matrix binding are ranked based on their absolute value of log fold‐change (FC). A total of 50, 52 and eight genes are differentially expressed out of 1183, 859 and 49 genes, respectively. All the DEGs that are annotated to extracellular space are ranked based on their absolute value of log FC. The top genes are shown. Upregulated genes are shown in red, and downregulated genes are shown in blue. The box and whisker plot on the left summarises the distribution of all the DEGs that are annotated to this GO term. (C) Ancestor charts for top identified biological processes, cellular components and molecular functions impacted by Sel‐GemPac treatment in KPC mice. The regulation of cell population proliferation regulates biological processes via cellular process and biological regulation. Extracellular space acts via the cellular anatomical entity. The extracellular matrix binding directly modulates molecular functions.

iPathwayGuide is used to compare the number of DEGs annotated to each GO term versus the expected number of DEGs occurring just by chance.^20^, ^21^ The method uses an over‐representation approach for computing the statistical significance with FDR. A total of 1481 GO terms were found to be significantly enriched before the correction for multiple comparisons. Following an FDR correction, there were several hundred significantly enriched GO terms. The top listed GO terms involved in biological processes, cellular components and molecular functions are shown in Figure 4A. Under biological process‐associated GO term ‘regulation of cell population proliferation’, there were 1183 genes, in which 50 genes were found to be differentially expressed (21 upregulated and 29 downregulated) in the cancer cells of treated KPC mice (Figure 4B and Table S4). The box and whisker plot summarised the distribution of all the DEGs, in which the median is skewed towards downregulation since there were more significant under‐expressed genes. Such polarisation indirectly suggests a negative impact on the particular pathway upon Sel‐GemPac treatment. The GO term ‘extracellular space’ falls under cellular components and involves 859 genes. In Sel‐GemPac‐treated KPC mice cancer/tumour cells, a total of 52 genes out of 859 were differentially expressed (22 upregulated and 30 downregulated) indicating the modulation of extracellular space, limiting cancer cell growth or spread upon treatment (Figure 4B and Table S5). The top molecular function‐associated GO term was ‘extracellular matrix binding’. There were 49 genes under this term, in which eight genes were differentially expressed (three upregulated and five downregulated) in the cancer cells of treated KPC mice (Figure 4B and Table S6). The downregulated genes include important integrin protein Itga2, matrix protein ECM1 as well as basic adhesion protein BCAM, which may negatively regulate extracellular matrix upon Sem‐GemPac treatment. The ancestor charts for top identified biological processes, cellular components and molecular functions have been depicted in Figure 4C. The regulation of cell population proliferation has a pronounced effect on the biological process involved in cellular growth. Therefore, these results indicate that Sel‐GemPac suppresses tumour growth by negatively affecting several processes including pathways supporting extracellular space, extracellular matrix binding and cellular growth.

### 3.6 Digital spatial proteomic analysis identified a few differentially expressed proteins in Sel‐GemPac‐treated KPC mice tumour tissues

**FIGURE 5:**
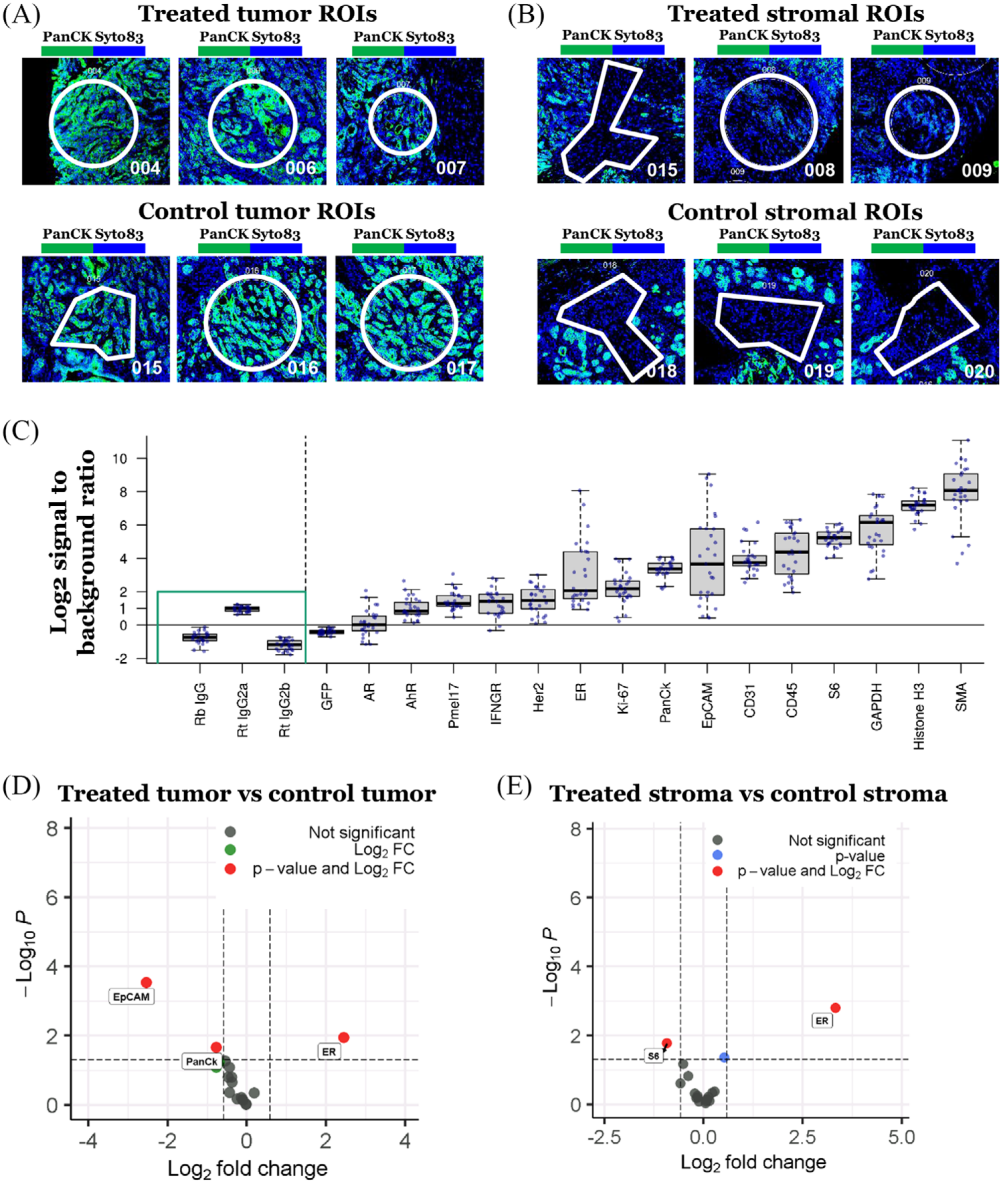
Digital spatial proteomic analysis of control and treated LSL‐Kras^G12D/+^; LSL‐Trp53^R172H/+^; Pdx1‐Cre (KPC) mice tumours. Processing of KPC mice tumour tissue sections for digital spatial profiling (DSP) analysis utilising GeoMx nanostring platform. Region of interests (ROIs, right panel) was selected in triplicates based on haematoxylin and eosin (H&E) staining and morphology markers. Complimentary photocleavable antibody‐oligo sequences were utilised for sequencing and detection. (A) Selected three ROIs from treated and control KPC mice tumours containing more than 50 cells. (B) Selected three ROIs from treated and control KPC mice stroma containing more than 50 cells. For the selection of specific cell types, circle, rectangle or irregular shapes were drawn around the regions. (C) The signal (log2) to background ratio plot for each protein target. Negative controls (IgGs) (green) are plotted on the far left of the plot. All the protein targets are above the baseline except green fluorescent protein (GFP) and androgen receptor (AR), which are close to the baseline. All these proteins were analysed for differential expression comparisons. (D) Volcano plot for the expressed proteins in treated KPC tumour cells compared to control KPC tumour cells. (E) Volcano plot for the expressed proteins in treated KPC stromal cells compared to control KPC stromal cells. In volcano plots, unadjusted *p*‐value of .05 and fold‐change (FC) of 1.5 were used to identify differentially expressed proteins. Non‐significant proteins, proteins with FC ≥1.5, proteins with *p*‐value <.05, and significant proteins are shown in grey, green, blue and red dots.

We also conducted a digital spatial proteomic analysis of KPC mice tumour tissue sections utilising GeoMx nanostring technology (Figure 5). The cancer cells or stromal cells in the treated and control tumour sections were selected specifically as ROI depending on the H&E staining and mouse Pan‐CK morphology marker in a similar manner as in the transcriptomic analysis (Figure 3). Three selected ROIs of cancer cells and stromal cells from the treated and control KPC mice tumours section containing more than 50 cells have been depicted in Figure 5A,B. The mouse pan‐tumour module from GeoMx nanostring platform was selected, which involves 19 proteins including controls. The signal (log2) to background ratio was determined for each protein target (Figure 5C). The proteins of the pan‐tumour panel that were found to be above the threshold included aryl hydrocarbon receptor (AHR), premelanosome protein 17 (PMEL17), interferon gamma receptor (IFNGR), human epidermal growth factor receptor 2 (HER2), estrogen receptor (ER), Ki‐67, PanCK, EpCAM, CD31, CD45 and ribosomal protein 6. The differential expression of these proteins in the cancer cells showed polarisation towards negative FC, however, only two of the proteins reached the level of significance. The PanCK and EpCAM showed significant downregulation in Sel‐GemPac‐treated cancer cells of KPC mice tumour (Figure 5D). The trends are similar for the stomal compartment. A downregulation of S6 protein was evident in the Sel‐GemPac‐treated stromal cells of KPC mice tumour (Figure 5E). For both cancer cells and stromal cells, the ER protein was found to be upregulated.

### 3.7 Two‐dimensional t‐SNE analysis of snRNAseq reveals the effect of Sel‐GemPac on cellular clusters in treated KPC mice tumour

**FIGURE 6:**
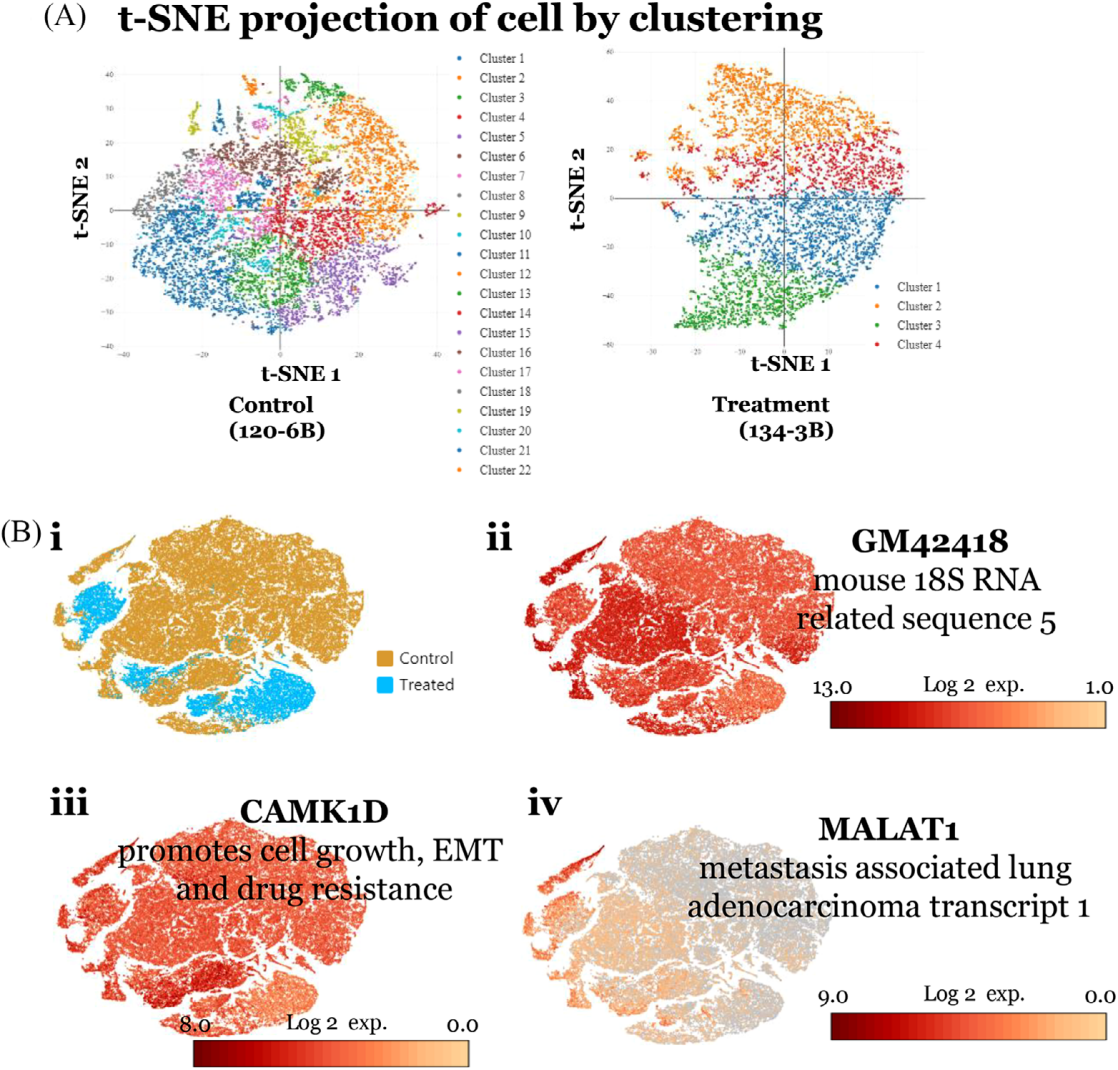
Two‐dimensional t‐distributed stochastic neighbour embedding (t‐SNE) combined analysis of single nuclear RNA sequences from untreated (control) and treated LSL‐Kras^G12D/+^; LSL‐Trp53^R172H/+^; Pdx1‐Cre (KPC) tumour cells. (A) Two‐dimensional t‐SNE analysis of single nuclear RNA sequences from representative control (120‐6B) and treated (134‐3B) KPC mice tumour cells. Different clusters of cells are colour‐coded. (B) (i) Merged clusters of control and treated KPC tumour cells. All clusters of control are in brown colour, whereas all clusters of treatment are in blue colour. Expression of *GM42418* (ii), *CAMK1D* (iii) and *MALAT1* (iv) among the clusters. Dark red indicates higher expression of transcript targets and colour key is shown below of each target.

**FIGURE 7:**
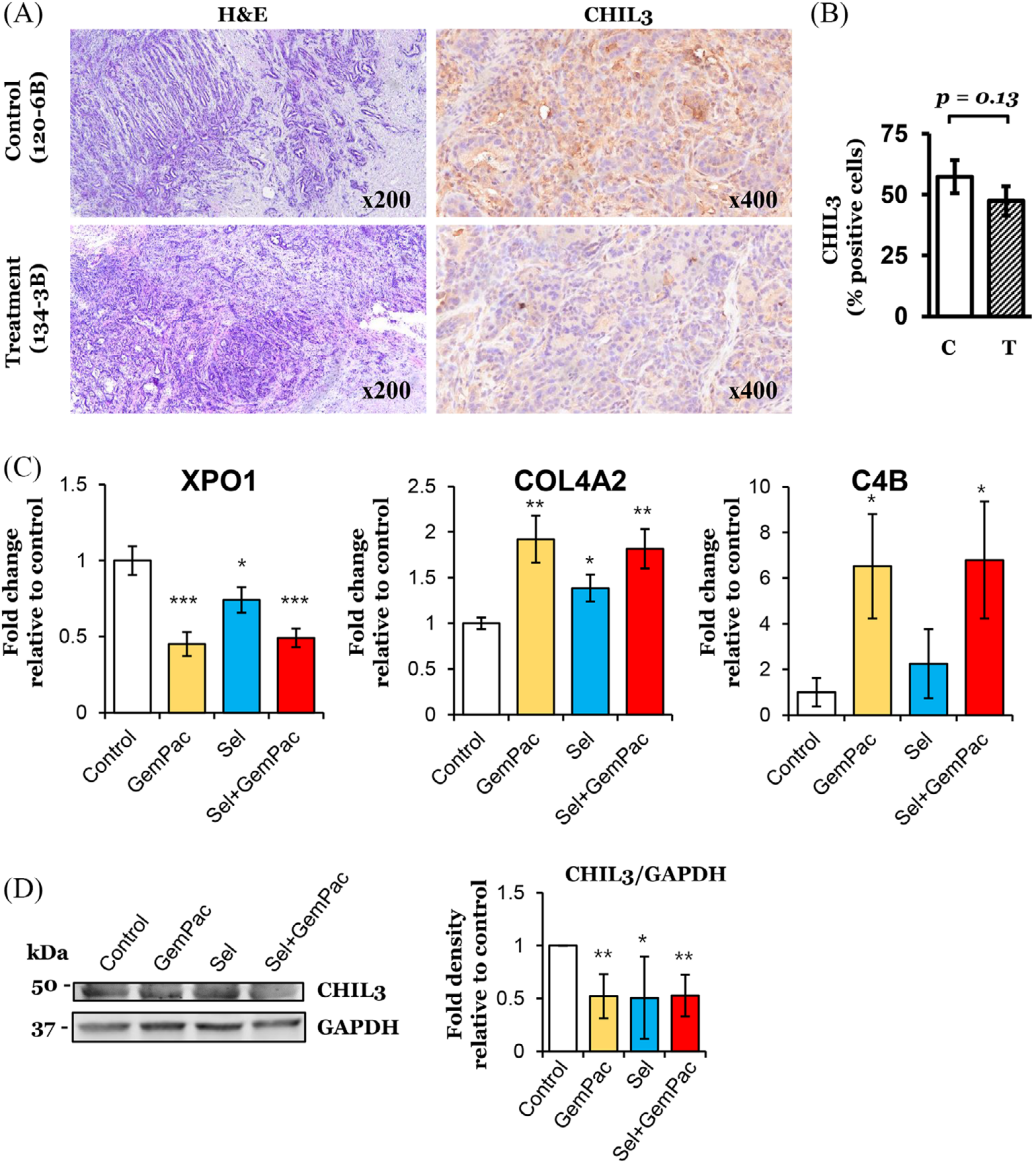
Validation of transcriptomic finding using immunohistochemical staining, quantitative polymerase chain reaction (qPCR) and immunoblot analysis. (A) Immunohistochemical staining of CHIL3 in representative control (120‐6B) and treated (134‐3B) tumour tissues, respectively. Corresponding haematoxylin and eosin (H&E) staining is shown in the left panel. (B) Percentage of CHIL3‐positive cells in the tissue samples. C, control; T, Sel‐GemPac treated. Original magnifications are shown on each histopathological image. (C‐D) Validation of expression of top differentially expressed genes in LSL‐Kras^G12D/+^; LSL‐Trp53^R172H/+^; Pdx1‐Cre (KPC) tumour‐derived KCI‐313 cell line using real‐time qPCR and immunoblot analysis. Cells were treated with gemcitabine (Gem) (20 nM), paclitaxel (Pac) (15 nM) and selinexor (Sel) (400 nM) for 24 h or 72 h. Sel‐GemPac, selinexor–gemcitabine–nab‐paclitaxel.

To explore the effect of Sel‐GemPac on the tumour microenvironment and on the bulk tumour, snRNAseq analysis was performed both for untreated and treated murine PDAC KPC tumours. The 10× Genomics Chromium single cell RNA‐Seq platform was utilised for this purpose. After quality control of single nuclear transcriptomic profiling and subsequent analysis, we observed a reduced number of clusters in Sel‐GemPac‐treated KPC tumours (Figures 6A and S4). The composition of tumours was determined by applying 2D t‐SNE analysis, and a total of 17 and 22 clusters were observed in treatment naïve KPC tumours, whereas it was reduced to 4 and 7 clusters in Sel‐GemPac‐treated KPC tumours. A combined colour‐coded cluster distribution of control and treated KPC mice tumours is shown in Figure S5. Among different cell types, reduced expression of CD44+ stem cells, platelet and endothelial cell adhesion molecule 1 (*PECAM1*)‐positive endothelial cells were noted in the drug treatment group (Figure S6). These results indicate a pronounced effect of Sel‐GemPac therapy in the murine PDAC KPC tumours. Further molecular analysis showed that Sel‐GemPac treatment caused suppression of calcium/calmodulin‐dependent protein kinase 1 D (CAMK1D), long non‐coding RNA (lncRNA) GM42418 and MALAT1 in most of the clusters of treated KPC tumour cells (Figure 6B). The top upregulated and downregulated genes are listed in Tables S7 and S8, respectively. These changes involve several clusters most importantly clusters 1, 5 and 28 (Tables S9–S11). Therefore, the results from snRNAseq clearly demonstrated the impact of Sel‐GemPac in the reduction of cellular clusters in the KPC mice‐derived tumours. Though functional identification of clusters was not possible, we have listed the most significantly altered genes in the topmost clusters (Table S12). These clusters may be considered as sub‐types of tumour cells itself suggesting intra‐tumour heterogeneity in the untreated KPC tumours. Next, we validated spatial transcriptomic findings in KPC mice‐derived cell line KCI‐313 using IHC and real‐time qPCR (Figure 7). Expression of CHIL3 was low in the Sel‐GemPac‐treated KPC mice tissue. IHC showed a trend of downregulation of CHIL3 in the treated tumour even though not reaching statistical significance (*p* = .13) (Figure 7A,B). The qPCR data revealed an upregulation of COL4A2 and C4B, which is in agreement with the DSP finding (Figure 7C). Interestingly, we observed downregulation of XPO1 with GemPac and Sel both suggesting a novel mechanism of XPO1 deactivation by GemPac. The immunoblot analysis confirmed downregulation of CHIL3 in the KPC cell line with drug treatment (Figure 7D). It seems both GemPac and Sel have a role in the downregulation of CHIL3.

## 4 DISCUSSION

Novel molecularly driven treatment strategies are urgently needed for PDAC patients that universally show limited benefits from GemPac therapy. SINE compound Sel can work synergistically with GemPac in human PDAC cell lines as well as the KPC mice tumour‐derived cell lines, however, the global impact of Sel‐GemPac in pancreatic tumours and microenvironment poorly understood.^9^ This is the first study to thoroughly characterise the impact of nuclear export inhibition‐based regimen at the molecular level in the genetically modified KPC mouse model through DSP and snRNAseq. Our DSP analysis showed a decrease in tumour promoting genes both in the cancer cells and the stromal compartment. The snRNAseq analysis demonstrated a significant reduction of cellular clusters in the Sel‐GemPac‐treated tumours. Collectively, these results demonstrated a broad penetration of Sel‐GemPac in the PDAC‐supporting signalling networks in the KPC mouse model.

Our previous research has demonstrated that the XPO1 is a viable therapeutic target in PDAC, and the SINE Sel can enhance the effectiveness of GemPac preclinically and a recommended phase II dose (RP2D) is being determined in a phase I study (NCT02178436).^9^ The synergy (CI < 1) is achieved at pharmacologically relevant concentrations and shows pronounced effect both in 2D colony and 3D spheroid formation. The efficacy of Sel‐GemPac at low concentrations further supports its applicability in clinical settings. Besides cellular and xenografts models, the genetically modified KPC mouse model has long been an important tool for preclinical testing of new therapeutic agents due to its close resemblance with human PDAC in terms of histology, genetics and response to therapy.^22^ It is encouraging to note that in our current study, the Sel‐GemPac‐treated KPC mice have exhibited an increase in median survival by over a month. To the best of our knowledge, this is the first study demonstrating the survival advantage of Sel‐GemPac in the KPC mouse model (*p* = .047). This finding implies that the combination treatment of Sel‐GemPac could potentially offer benefits to patients with PDAC.

The nuclear export mechanism is a universal transport system valid in tumour and other cells of the tumour microenvironment. Blocking XPO1 by Sel is bound to have a broad impact on nuclear retention‐mediated signalling in tumour and stromal cell compartments. However, to capture such changes requires departure from a single pathway to holistic approaches as demonstrated in this article. Therefore, we first investigated the microenvironment of Sel‐GemPac‐treated KPC mice tumours through IHC, DSP and snRNAseq. In IHC analysis, the proliferative marker Ki‐67 was found to be reduced, suggesting an overall anti‐proliferative effect in the treated KPC tumour tissue. We also observed nuclear accumulation of FOXO3a in treated tissue samples suggesting an activation of this protein. Several studies have considered the role of FOXO3a as a tumour suppressor in PDAC. Its downregulation has been linked to the activation of oncogenic signalling pathways, such as the PI3K/AKT and mitogen‐activated protein kinase kinase/extracellular signal‐regulated kinase (MEK/ERK). Besides PDAC cells, the tumour microenvironment has been shown to be regulated by FOXO3a via cytokines and chemokine‐mediated immune cells recruitment. Sunters et al. demonstrated that Pac induces the expression of FOXO3a in breast cancer, and this upregulation of FOXO3a results in an increase in BIM levels, ultimately causing apoptosis in the cancer cells.^23^ Similarly, the presence of Pac in Sel‐GemPac combination might be the contributing factor for the robust expression of FOXO3a observed in our study. Our group has previously shown nuclear retention of FOXO3a in the clinical trial biopsies of solid tumor patients treated with Sel.^24^ In PDAC, collagen fibres usually accumulate in the extracellular matrix surrounding the tumour cells. This desmoplastic microenvironment is a hallmark of PDAC and contributes to its aggressiveness. The cancer cells stimulate the production of collagen by cancer‐associated fibroblasts, which can lead to a number of negative effects such as tumour stiffness, increased interstitial pressure, vascular collapse and enhanced mechanosignalling.^25^, ^26^, ^27^ Such rigidity hinders drug penetration and infiltration of immune cells, resulting in chemotherapy resistance and immunosuppressive microenvironment.^28^ Interestingly, Sel‐GemPac treatment resulted in loosening of the stroma (Figure 4C), indicating penetrance to the tumour site by this drug combination.

To study the tumour–microenvironment interactions in the KPC tumour within its native morphological context, we took the advantage of nanoString GeoMx DSP technology. The DSP analysis unveiled a decrease in molecules that promote tumour growth, such as CHIL3, EpCAM, MMP7 and several pathways, including CAM and PI3K‐AKT signalling pathway. Previous studies have reported that CHIL3 is a tumour‐associated macrophage (TAM) differentiation marker.^29^ Furthermore, it has been demonstrated that CHIL3 can regulate immune function in various diseases including PDAC^30^, ^31^ and robust downregulation of CHIL3 may have tumour suppressing effects. Epithelial cellular adhesion molecule (EpCAM) is also frequently overexpressed in PDAC. EpCAM has been proposed to be involved in proliferation, survival, invasion, migration and stemness.^32^ EpCAM carries out such diverse functions due to its influence on AKT and ERK signalling and several MMPs. Therefore, EpCAM is considered as a potential therapeutic target for PDAC. In our current study, Sel‐GemPac treatment not only inhibited the EpCAM transcript, but also suppressed the protein expression significantly in KPC mice tumour. Another important molecule that is downregulated in drug‐treated KPC tumour tissue is MMP7. MMP7 is found to be involved in tumour invasion, metastasis, angiogenesis and inflammatory processes.^33^, ^34^ Many gastrointestinal malignancies demonstrate overexpression of MMP7 including cancer of the pancreas. Overexpressed MMP7 exerts its effect through ECM degradation via activation of MMP2 and MMP9.^33^ Collectively, the presented DSP findings suggest a strong tumour suppressive effect of Sel‐GemPac in the cancer cell compartment as well as stromal compartment of spontaneous genetically modified KPC mouse tumour. The DSP of pan‐cancer protein panel also showed a trend towards downregulation of most cancer‐associated proteins. However, upregulation of ER was consistent both in cancer cells and stroma of KPC tumours. The significance of ER expression in PDAC remains largely unknown.^35^, ^36^, ^37^ There are studies that have demonstrated that the G protein‐coupled estrogen receptor can inhibit PDAC via activation of ER^36^ suggesting one of the important roles of ER in PDAC.

Several studies have reported cellular heterogeneity utilising single cell RNA‐seq earlier focusing on untreated KPC tumours only.^38^, ^39^, ^40^ Such studies observed normal or tumour ductal cell types, fibroblasts and different immune cells infiltrations.^38^, ^39^ In our study, we have observed the presence of CD44‐positive stem cells in the untreated KPC tumours, which is greatly reduced in the Sel‐GemPac‐treated tumours. The CD44‐positive stem cells are associated with Gem or chemotherapy resistance in PDAC.^41^, ^42^ A limited infiltration of immune cells such as CD3+, CD4+, CD8+, CD19+ cells and Ly6C1 macrophages (Figure S6) were observed in the current study, both in untreated and treated KPC tumours. This might be due to the harvesting of late‐stage KPC tumours as observed by other independent groups.^40^ Though CD4 expressing cells were rarely detected both in untreated and treated KPC tumour's snRNA‐seq, we observed CD4+ cells in untreated tumours using IHC staining. Importantly, the expression was decreased in the treated tumour tissues (Figure S7). The lower detection rate in our snRNAseq may be due to multiple factors. Isolation of single cells involves a number of digestion processes and nucleus isolation requires additional steps. It is possible that some cells may have been lost during the isolation of single cells via multiple digestion cycles.^40^

Our snRNA‐seq analysis showed that Sel‐GemPac treatment was significantly effective in reducing the expression of *CAMK1D*, lncRNA *GM42418* and *MALAT1* genes. The calcium/calmodulin‐dependent protein kinase (CAMK) has been shown to regulate a diverse range of cancer‐related functions in different tumour types.^43^ In 2021, the role of CAMK1 was demonstrated in PDAC.^44^ The study found higher expression of CAMK1 in pancreatic cancer from bioinformatics as well as TMA‐IHC analyses. The protein‐‐protein interactions network predicted an association of CAMK1 with a number of genes including *CALM1*, *CALM3*, *CREB1*, *CALM2*, *SYN1*, *NOS3*, *ATF1*, *GAPDH*, *PPM1F* and *FBXL12*. Most of these genes are involved in aldosterone synthesis and secretion, oxytocin signalling pathways and are upregulated in PDAC. The role of *MALAT1* in pancreatic cancer aggressiveness is well documented^45^, ^46^, ^47^ due to its involvement in *KRAS* expression regulation.^48^ These observations indicate an important role of *CAMK1D* genes and *GM42418*, *MALAT1* lncRNAs in the regulation of tumour growth in KPC genetic mouse model. Our single clear nuclear RNA analysis has identified a novel less recognised lncRNA. The role of *GM42418* has not yet been demonstrated in pancreatic cancer and can be subjected to further investigation. Among the top differentially expressed transcripts, some such as CHIL3, COL4A2 and C4B observed in DSP have been validated using IHC or qPCR. There are some limitations of this study. Though demonstrated in our earlier publication, however, due to the rarity and management of large KPC mouse colony current study lacks GemPac only and Sel only groups. The study also utilised only two control and two treated tumours for the snRNAseq and DSP analysis that requires further validation.

In conclusion, our results show that Sel‐GemPac‐mediated inhibition of tumour growth is through broad impact on several signalling mechanisms supporting PDAC tumour and stromal microenvironment compartments. These findings provide support for the understanding of the molecular mechanism of Sel‐GemPac‐mediated inhibition of PDAC growth. Further investigations on the utility of the tumour and microenvironmental targets identified as potential biomarkers of response to Sel‐GemPac in clinical studies are warranted.

## AUTHOR CONTRIBUTIONS

Asfar S. Azmi designed the study, interpreted the results, analysed the data and wrote and edited the paper. Md. Hafiz Uddin, Husain Yar Khan, and Sahar F. Bannoura performed in vitro experiments and wrote and edited the paper. Amro Aboukameel performed the KPC mice experiment. Yiwei Li performed human tissue analysis. Gregory Dyson and Seongho Kim performed bioinformatics and statistics. Mohammad Najeeb Al‐Hallak, Yosef Mzannar, Ibrahim Azar, Tanya Odisho, Amr Mohamed,Yosef Landesman, Steve Kim, Rafic Beydoun, Philip A. Philip and Anthony F. Shields wrote and edited the manuscript. Ramzi M. Mohammad analysed the animal studies and wrote and edited the manuscript. All authors provided significant scientific input in this study and wrote and edited the paper. All authors are aware of the content of this paper.

## CONFLICT OF INTEREST STATEMENT

Asfar S. Azmi is a council member at GLG and Guidepoint. Yosef Landesman was an employee of Karyopharm Therapeutics Inc. All other authors declare they have no conflicts of interest.

## ETHICS STATEMENT

This study was approved by the animal ethics committee of Wayne State University. Animal care and experiments were conducted in compliance with the Institutional Animal Care and Use Committee and NIH guidelines.

## Supporting information

Supporting Information

Supporting Information

Supporting Information

Supporting Information

Supporting Information

Supporting Information

Supporting Information

Supporting Information

Supporting Information

Supporting Information

Supporting Information

Supporting Information
