# Supplementary material for: Molecular analysis of XPO1 inhibitor and gemcitabine–nab‐paclitaxel combination in KPC pancreatic cancer mouse model
Source: Clin Transl Med. 2023 Dec 22;13(12):e1513. doi: 10.1002/ctm2.1513 (PMC10739156; doi:10.1002/ctm2.1513)
Supplement: Supplementary file 1 — Supporting Information [file CTM2-13-e1513-s001.docx]

**Molecular analysis of XPO1 inhibitor and gemcitabine-nab-paclitaxel combination in KPC pancratic cancer mouse model**

Md. Hafiz Uddin^1^, Mohammad Najeeb Al-Hallak^1^, Husain Yar Khan^1^, Amro Aboukameel^1^, Yiwei Li^1^, Sahar F. Bannoura^1^, Gregory Dyson^1^, Seongho Kim^1^, Yosef Mzannar^1^, Ibrahim Azar^1^, Tanya Odisho^2^, Amr Mohamed^3^, Yosef Landesman^4^, Steve Kim^1^, Rafic Beydoun^1,5^, Ramzi M. Mohammad^1^, Philip A. Philip^6^, Anthony F. Shields^1^, Asfar S. Azmi^1^*

**Supplementary methods:**

**Single cell RNA-seq analysis**

The Seurat package in the R programming environment was sued to process the single-cell RNA sequencing (sc-RNA-seq) data. The number of cells measured for the 2 samples subjected to sc-RNA-seq were 7,668 and 4,607 before any filtering. To ensure the quality of the data, we applied the following filters to these data: cells had to have at least 50 genes detected, the percent mitochondrion in each cell had to be <20% and the percent of the largest gene count within each cell had to be <40%. In the end, these filters resulted in 1643 and 961 valid cells in our two experiments. That resultant data was normalized using the centered log ratio transformation method and subsequently scaled before dimension reduction using principal component analysis and an elbow plot to identify the appropriate number of dimensions, 8 in this case. We then identified the appropriate number of clusters in our data using the default settings from the ‘FindClusters’ algorithm [which follows the procedure from Waltman and van Eck, 2013^1^] from the Seurat package.

References:

1. Waltman and van Eck (2013) A smart local moving algorithm for large-scale modularity-based community detection. Eur. Phys. J. B (2013) 86: 471.

**Supplementary figure legends:**

**Supplementary figure S1.** Characterization of KPC mice derived KCI-313 cell line. A. Phase-contrast image of the cell line. B. Confirmation of KRAS and p53 mutation and presence of Cre. C. *Mycoplasma* spp. PCR.

**Supplementary figure S2.** Validation of KPC mice and PDAC tumor. (A-B) Confirmation of Pdx1-Cre introduction and mutations of KRAS and p53 in the KPC mice both in control and Sel-GemPac treated group using conventional PCR and subsequent 2% agarose gel electrophoresis. DNA was isolated from the ear of mice (collected using ear punch) using commercially available kit. (C) Ultrasonographic evaluation of suspected tumors in the KPC mice. Suspected tumors are shown as spheres of magenta color. The pancreas, kidney, aorta, and splenic vessels are also depicted in the sonograph. (D) Hematoxylin and Eosin (H & E) staining of pancreatic tumor tissue showing desmoplastic changes (indicated with red arrow in the left) confirming pancreatic ductal adenocarcinoma (PDAC, indicated with red arrow) in representative mouse. Specific area highlighting adenocarcinoma shown in 200x magnification on the right.

**Supplementary figure S3.** Digital spatial transcriptomics (DSP) analysis of control and treated KPC mice stroma. (A) Selected three ROIs from treated and control KPC mice tumors containing more than 50 cells. For the selection of specific cell types, circle, rectangle or irregular shapes were drawn around the regions. (B) Differentially expressed genes (DEGs) between treated and untreated KPC mice tumor sections. The dotted lines are used to select the DEGs. Significantly (*p* < 0.05) overexpressed genes were shown in red and under-expressed genes were shown in blue. (C) Top impacted pathways in the treated tumors compared to control tumors. The x-axis indicated over-representation (pORA) and the y-axis indicated total pathway accumulation (pAcc). Each dot represents a pathway and dot size is proportional to the represented pathway. Significant and non-significant pathways were shown in red and black respectively. (D-F) Impacted genes and pathways associated with transcriptional misregulation, relaxin and PPAR signaling. The genes that show differential expression are arranged according to their log fold change. Genes that have been upregulated are visualized in red, while genes that have been downregulated are represented in blue. On the left, the box and whisker plot provide a summary of the distribution of all genes. The box in the plot depicts the first quartile, median, and third quartile of the distribution, whereas any outliers in the data are depicted as circles. Each gene's computed perturbation is overlaid on the pathway diagram. The perturbation considered both the measured fold change of each gene and the accumulated perturbation propagated from any upstream genes, accounting for the cumulative effect on downstream genes. The highest negative and positive perturbations are shown in dark blue and dark red respectively.

**Supplementary figure S4.** Two-dimensional t-distributed stochastic neighbor embedding (t-SNE) analysis of single nuclear RNA sequences from an untreated (left, 117-6B) and a treated (right, 136-6B) KPC tumor cells. Different clusters of cells are color-coded and shown on the right of each t-SNE image.

**Supplementary figure S5.** Two-dimensional t-distributed stochastic neighbor embedding (t-SNE) combined analysis of single nuclear RNA sequences from all untreated (top; 117-6B, 120-6B) and treated (bottom; 134-6B, 136-6B) KPC tumor cells. Different clusters of cells are color-coded and shown on the right of the image.

**Supplementary figure S6.** Two-dimensional t-distributed stochastic neighbor embedding (t-SNE) combined analysis of single nuclear RNA sequences from all untreated (control) and treated KPC tumor cells. (A) Merged clusters of control and treated KPC tumor cells. (B) Clusters containing positive CD44 stem cells. The log2 expressions of CD44 stem cell marker is shown in color gradients. (C) Clusters containing positive markers for other cell types including endothelial, fibroblast and immune cells.

**Supplementary figure S7.** Immunohistochemical staining of KPC tumors for CD4 from untreated and treated mice along with H&E staining. Original magnifications are shown on each histopathological image.

**Supplementary table legends**

**Supplementary table S1.** List of primers and sequences used for RT-qPCR.

**Supplementary table S2.** Differentially expressed genes between cancer cell ROIs of treated and control KPC tissue samples.

**Supplementary table S3.** Differentially expressed genes between stromal cell ROIs of treated and control KPC tissue samples.

**Supplementary table S4.** Differentially expressed genes in biological processes associated GO term regulation of cell population proliferation.

**Supplementary table S5.** Differentially expressed genes in cellular component associated GO term extracellular space.

**Supplementary table S6.** Differentially expressed genes in molecular function associated GO term extracellular matrix binding.

**Supplementary table S7.** Top downregulated genes in the treated KPC mouse compared to control. *, *p* < 0.05; **, *p* < 0.01; ***, *p* < 0.001.

**Supplementary table S8.** Top upregulated genes in the treated KPC mouse compared to control. *, *p* < 0.05; ***, *p* < 0.001.

**Supplementary table S9.** Log2 fold change in cluster 1 between control and treated KPC mouse tumor. Adjusted *p-*values are in the last column.

**Supplementary table S10.** Log2 fold change in cluster 5 between control and treated KPC mouse tumor. Adjusted *p-*values are in the last column.

**Supplementary table S11.** Log2 fold change in cluster 28 between control and treated KPC mouse tumor. Adjusted *p-*values are in the last column.

**Supplementary table S12.** Log2 fold change of significantly differentially expressed genes in topmost clusters observed in snRNAseq data.
