# Supplementary material for: Molecular analysis of XPO1 inhibitor and gemcitabine–nab‐paclitaxel combination in KPC pancreatic cancer mouse model
Source: Clin Transl Med. 2023 Dec 22;13(12):e1513. doi: 10.1002/ctm2.1513 (PMC10739156; doi:10.1002/ctm2.1513)
Supplement: Supplementary file 2 — Supporting Information [file CTM2-13-e1513-s012.pptx]

## Slide 1
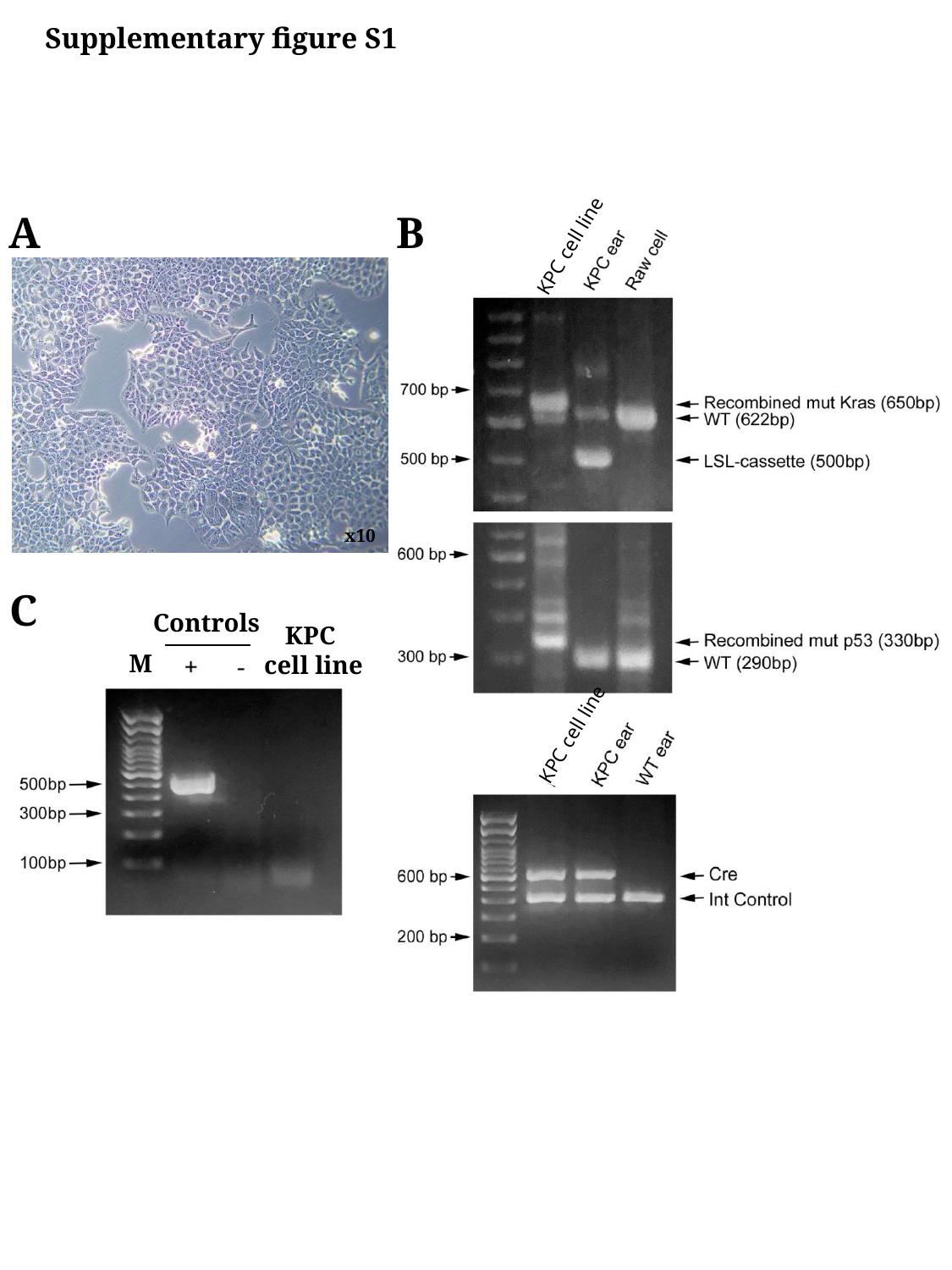

Supplementary figure S1
KPC cell line
A
B
x10
C
Controls
KPC
cell line
M
KPC cell line

## Slide 2
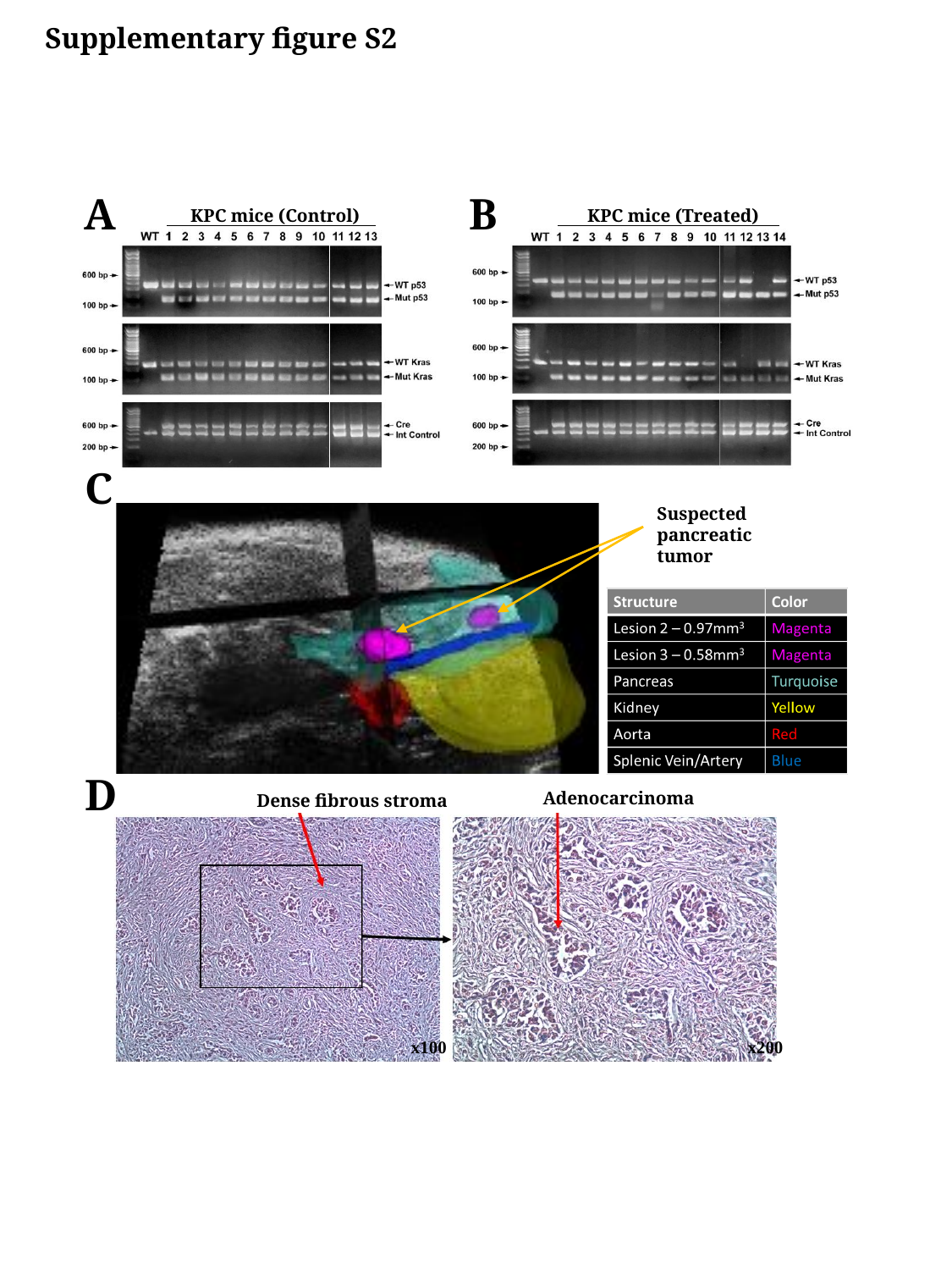

Supplementary figure S2
A
B
KPC mice (Control)
KPC mice (Treated)
C
Suspected
pancreatic
tumor
D
Adenocarcinoma
Dense fibrous stroma
x100
x200

## Slide 3
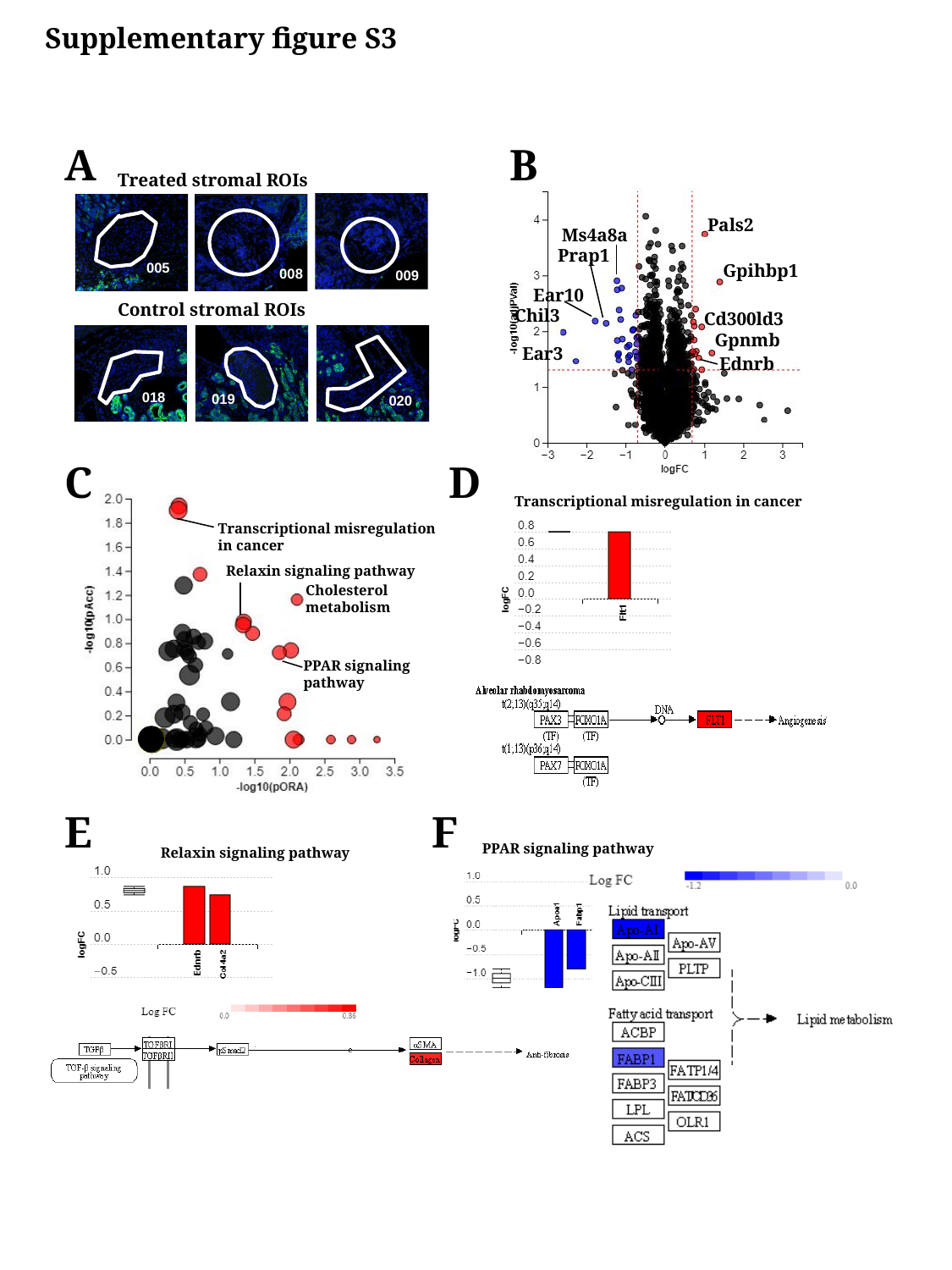

Supplementary figure S3
A
B
Treated stromal ROIs
Pals2
Ms4a8a
Prap1
Gpihbp1
Ear10
Chil3
Cd300ld3
Gpnmb
Ear3
Ednrb
009
005
008
Control stromal ROIs
019
020
018
C
D
Transcriptional misregulation in cancer
Relaxin signaling pathway
Cholesterol metabolism
PPAR signaling pathway
Transcriptional misregulation in cancer
E
F
PPAR signaling pathway
Relaxin signaling pathway

## Slide 4
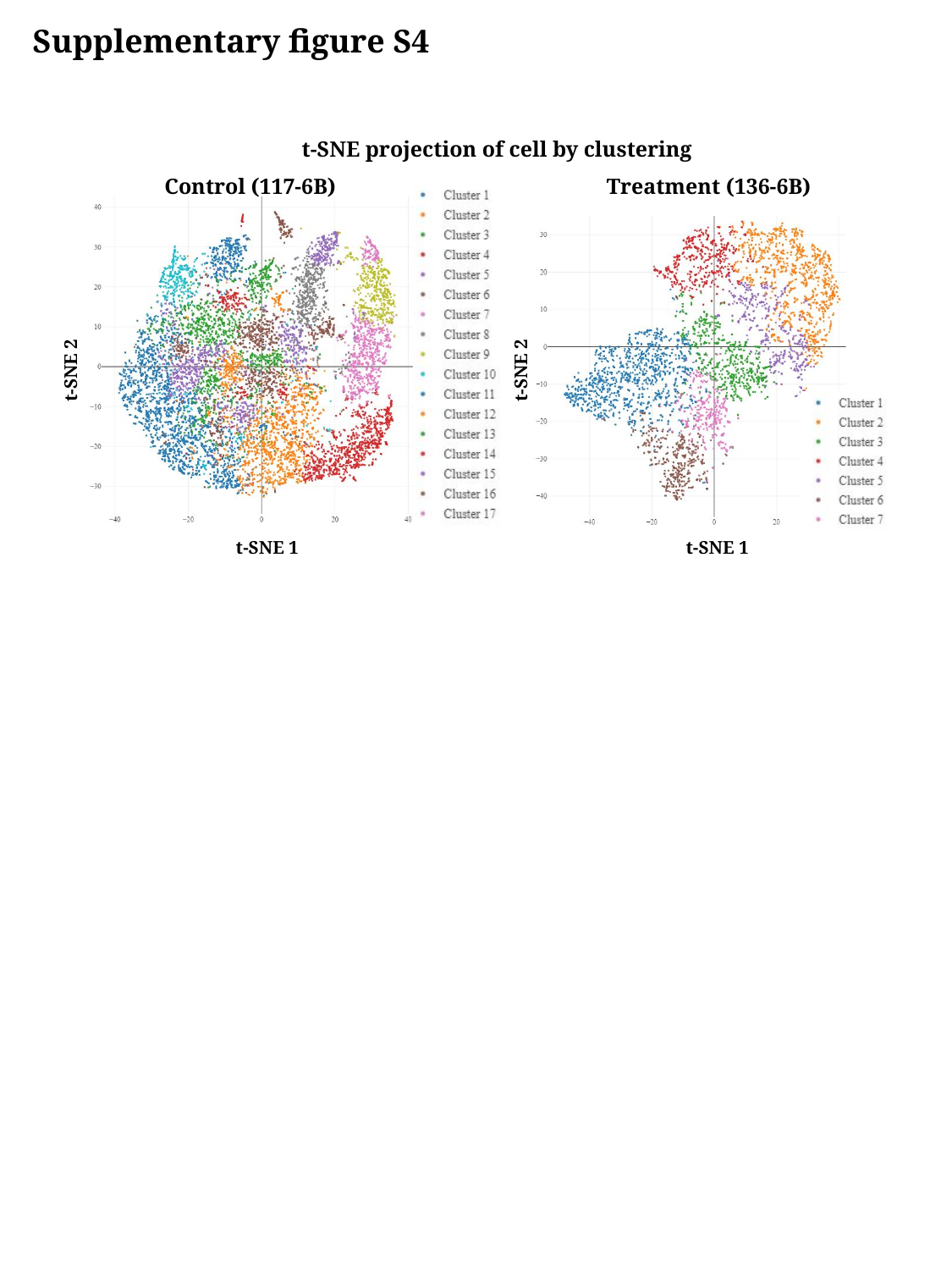

Supplementary figure S4
t-SNE projection of cell by clustering
Control (117-6B)
Treatment (136-6B)
t-SNE 2
t-SNE 2
t-SNE 1
t-SNE 1

## Slide 5
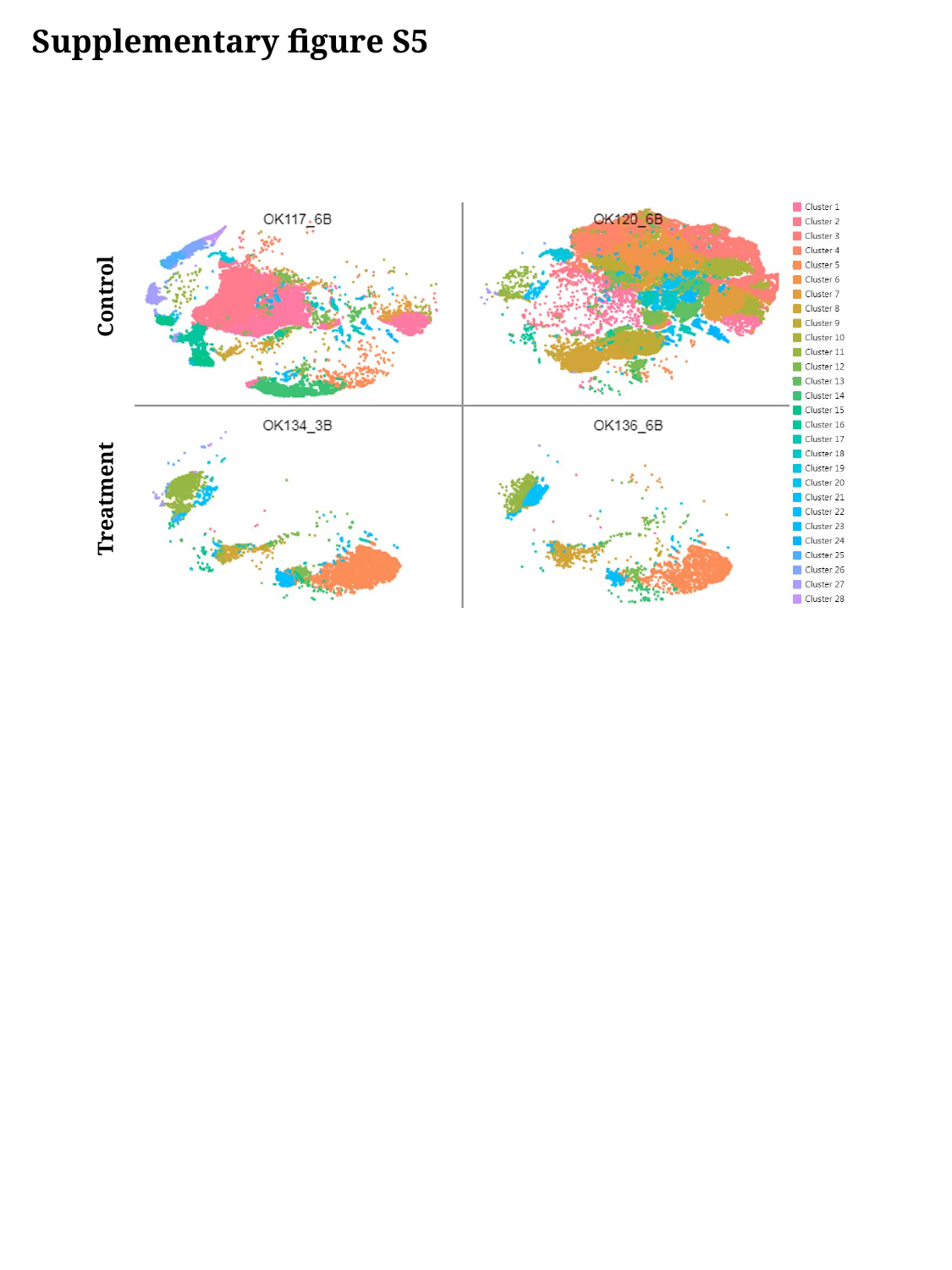

Supplementary figure S5
Control
Treatment

## Slide 6
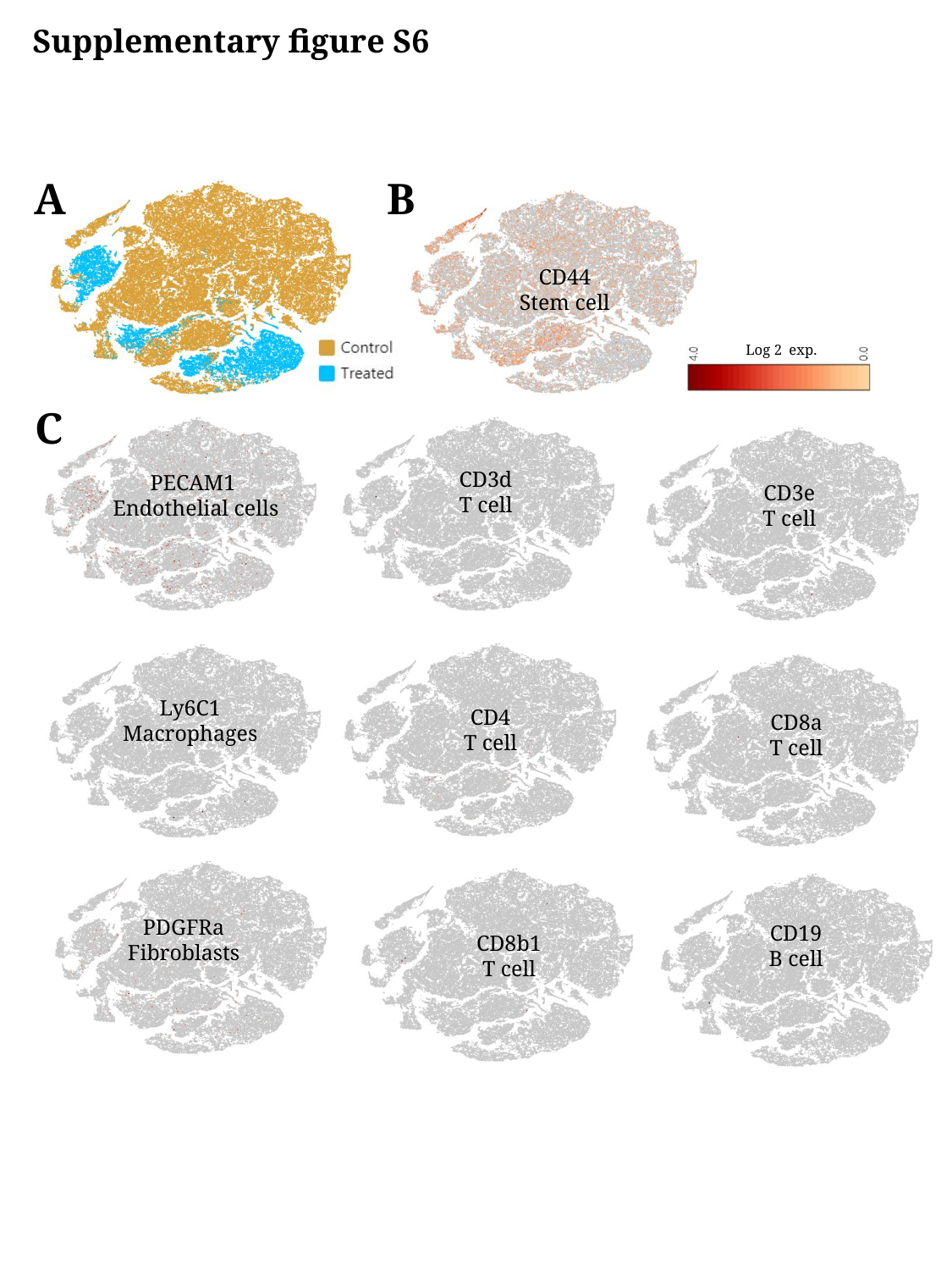

Supplementary figure S6
A
B
CD44
Stem cell
C
CD3d
T cell
PECAM1
Endothelial cells
CD3e
T cell
Ly6C1
Macrophages
CD4
T cell
CD8a
T cell
PDGFRa
Fibroblasts
CD19
B cell
CD8b1
T cell
Log 2 exp.

## Slide 7
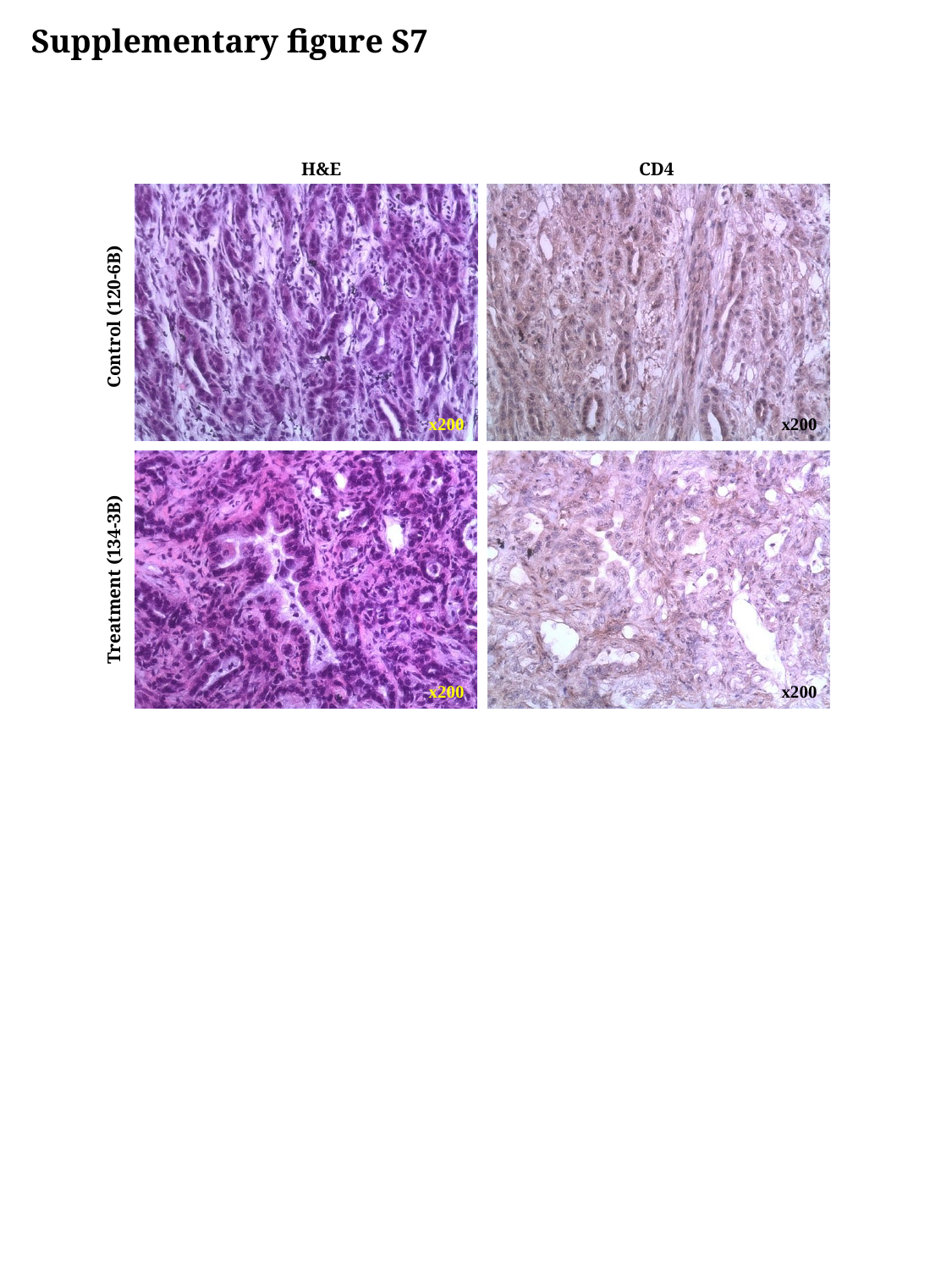

Supplementary figure S7
H&E
CD4
Control (120-6B)
x200
x200
Treatment (134-3B)
x200
x200
