# Supplementary material for: Molecular analysis of XPO1 inhibitor and gemcitabine–nab‐paclitaxel combination in KPC pancreatic cancer mouse model
Source: Clin Transl Med. 2023 Dec 22;13(12):e1513. doi: 10.1002/ctm2.1513 (PMC10739156; doi:10.1002/ctm2.1513)
Supplement: Supplementary file 3 — Supporting Information [file CTM2-13-e1513-s005.docx]

**Table S1. List of primers and sequences used for RT-qPCR.**

| **Name of the primers** | **Direction** | **Sequences (5’-3’)** |
| --- | --- | --- |
| Xpo1 | Forward | GGAAAACTGTGAAACCCACCTT |
|  | Reverse | GCTGCATGGTCTGCTAACAT |
| Col4a2 | Forward | GACCGAGTGCGGTTCAAAG |
|  | Reverse | CGCAGGGCACATCCAACTT |
| C4b | Forward | ACTTCAGCAGCTTAGTCAGGG |
|  | Reverse | GTCCTTTGTTTCAGGGGACAG |
| Gapdh | Forward | CGGGGTCCCAGCTTAGGTTC |
|  | Reverse | GGCCAAATCCGTTCACACCG |
