# Supplementary figures and images for: Molecular analysis of XPO1 inhibitor and gemcitabine–nab‐paclitaxel combination in KPC pancreatic cancer mouse model
Source: Clin Transl Med. 2023 Dec 22;13(12):e1513. doi: 10.1002/ctm2.1513 (PMC10739156; doi:10.1002/ctm2.1513)

## Slide 1
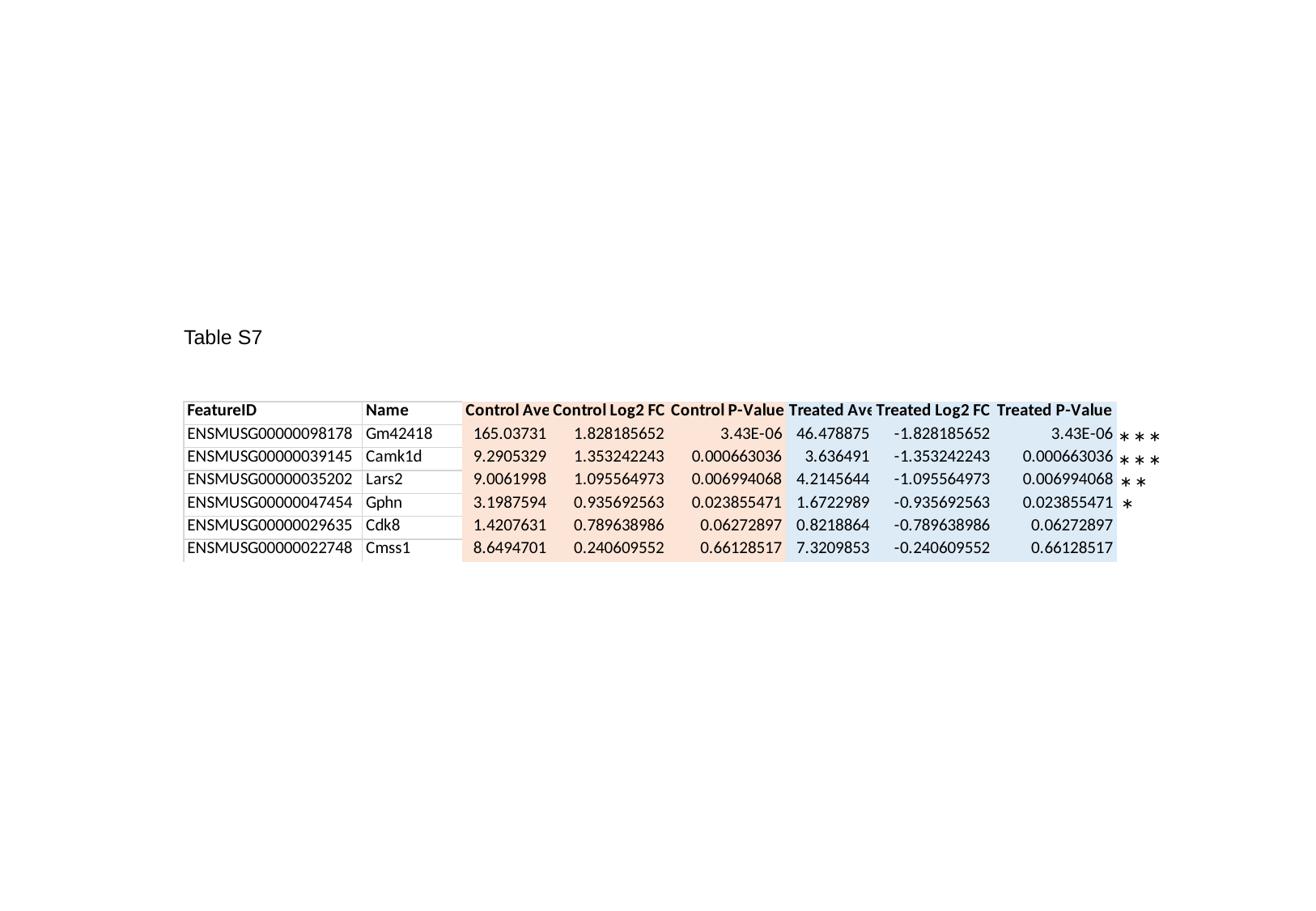

***
***
**
*
Table S7

Supplement: Supplementary file 9 — Supporting Information [file CTM2-13-e1513-s004.pptx]

## Slide 1
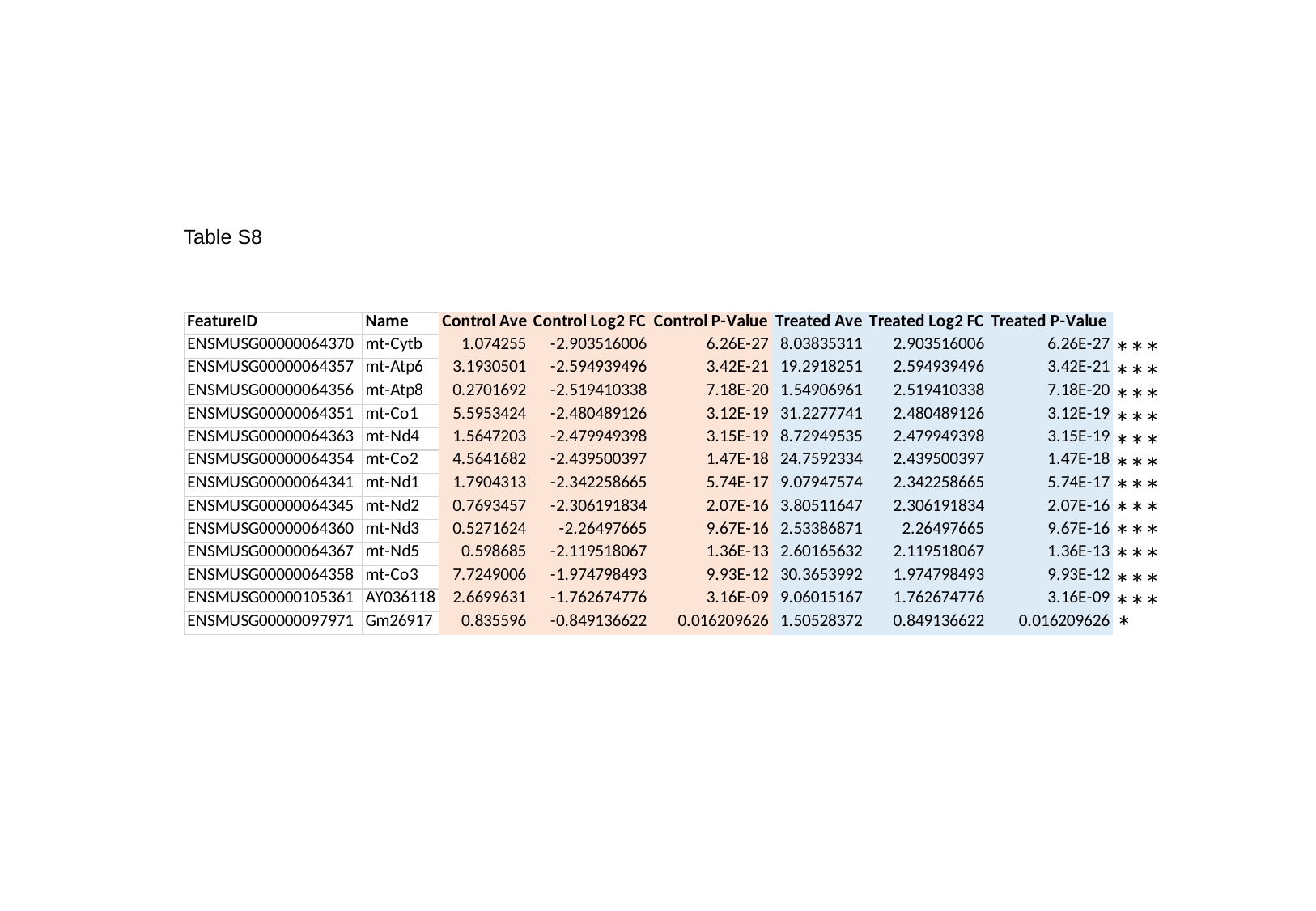

***
***
***
***
***
***
***
***
***
***
***
***
*
Table S8

Supplement: Supplementary file 10 — Supporting Information [file CTM2-13-e1513-s008.pptx]
