# Supplementary material for: Molecular analysis of XPO1 inhibitor and gemcitabine–nab‐paclitaxel combination in KPC pancreatic cancer mouse model
Source: Clin Transl Med. 2023 Dec 22;13(12):e1513. doi: 10.1002/ctm2.1513 (PMC10739156; doi:10.1002/ctm2.1513)
Supplement: Supplementary file 11 — Supporting Information [file CTM2-13-e1513-s006.docx]

**Table S9. Log2 fold change in cluster 1 between control and treated KPC mouse tumor. Adjusted *p*-values are in the last column.**

**DOI**: https://figshare.com/search?q=10.6084/m9.figshare.24481531

**Table S10. Log2 fold change in cluster 1 between control and treated KPC mouse tumor. Adjusted *p*-values are in the last column.**

**DOI**: https://figshare.com/search?q=10.6084/m9.figshare.24481699

**Table S11. Log2 fold change in cluster 1 between control and treated KPC mouse tumor. Adjusted *p*-values are in the last column.**

**DOI**: https://figshare.com/search?q=10.6084/m9.figshare.24481768
